# Nickel(II) Complex of N_4_ Schiff Base Ligand as a Building Block for a Conducting Metallopolymer with Multiple Redox States

**DOI:** 10.3390/molecules26092646

**Published:** 2021-04-30

**Authors:** Mikhail Karushev, Evgenia Smirnova, Irina Chepurnaya

**Affiliations:** Ioffe Physical-Technical Institute of the Russian Academy of Sciences (Ioffe Institute), 26 Politekhnicheskaya Str., 194021 St. Petersburg, Russia; Esmirnova@mail.ioffe.ru (E.S.); Ichepurnaya@mail.ioffe.ru (I.C.)

**Keywords:** metallocomplex, metallopolymer, Schiff base, redox properties, conducting properties, optical properties, energy storage, energy saving, charge delocalization, metal–ligand

## Abstract

Metal–ligand interactions in monomeric and polymeric transition metal complexes of Schiff base ligands largely define their functional properties and perspective applications. In this study, redox behavior of a nickel(II) N_4_-anilinosalen complex, [NiAmben] (where H_2_Amben = N,N′-bis(*o*-aminobenzylidene)ethylenediamine) was studied by cyclic voltammetry in solvents of different Lewis basicity. A poly-[NiAmben] film electrochemically synthesized from a 1,2-dichloroethane-based electrolyte was investigated by a combination of cyclic voltammetry, electrochemical quartz crystal microbalance, in situ UV-Vis spectroelectrochemistry, and in situ conductance measurements between −0.9 and 1.3 V vs. Ag/Ag^+^. The polymer displayed multistep redox processes involving reversible transfer of the total of ca. 1.6 electrons per repeat unit, electrical conductivity over a wide potential range, and multiple color changes in correlation with electrochemical processes. Performance advantages of poly-[NiAmben] over its nickel(II) N_2_O_2_ Schiff base analogue were identified and related to the increased number of accessible redox states in the polymer due to the higher extent of electronic communication between metal ions and ligand segments in the nickel(II) N_4_-anilinosalen system. The obtained results suggest that electrosynthesized poly-[NiAmben] films may be viable candidates for energy storage and saving applications.

## 1. Introduction

Sustainable energy has fast become the need of the century [1]. Learning how to store harvested renewable energy and use this energy efficiently is a crucial step to overcome the global energy crisis caused by limited reserves of fossil fuels. Sustainable energy involves various technologies out of which energy storage systems (e.g., supercapacitors, rechargeable batteries) [2] and energy saving technologies (e.g., low energy-consumption displays, smart windows) [3] are of utmost importance. The challenge of developing new materials to obtain more efficient energy storage and saving devices is often met through controlled modification of electrode surfaces. Electrodes for electrochemical energy storage are coated with conductive and highly capacitive materials capable of multi-electron transfer [4], whereas electrodes for energy saving systems are modified with materials that undergo significant color changes by electrochemically induced redox reactions, i.e., display electrochromic properties [5]. Substances possessing dual functionality attract special attention as they are suitable for electrode modification in both types of devices.

Conducting metallopolymers advantageously combining the electronic properties of transition metals with the processability and structural variety of polymeric organic scaffolds have been successfully applied in the areas of energy saving and storing [6,7]. The perspectives for practical application of a metallopolymer in sustainable energy systems are largely defined by the type, number, and stability of electrochemically available redox states as well as the efficiency of switching between them, which are in turn influenced by the extent of mixing of metal and ligand orbitals. Multiple redox states often enable advanced electrode modifying materials possessing high specific capacity and multi-color electrochromism.

Research studies over the past several decades have demonstrated that nickel(II) complexes of tetradentate N_2_O_2_ Schiff base (*salen*-type) ligands constitute attractive building blocks for the preparation of conducting metallopolymers for energy storage and saving applications. Nickel–*salen* polymers could be described as a three-dimensional mixed bonding structure composed of [Ni*salen*] units joined by different kinds of forces: covalent carbon–carbon bonds between *salen* moieties (usually in the *para* position of the phenyl rings) and non-covalent (intermolecular) interactions. Efficient electrochemical routes for the polymerization of the simplest representative of these compounds, N,N′-bis(salicylidene)ethylenediaminonickel(II) ([NiSalen]) and its analogues have been proposed. Charge transfer and storage, conductivity, and electrochromic properties of the obtained polymeric networks have been investigated, and the findings have been used to design polymer-modified electrodes for prototype devices. The details of the fundamental and applied studies in the area of nickel–*salen* metallopolymers can be found in several recently published reviews [8,9,10]. 

Poly-[Ni*salen*] films present an interesting case of metallopolymers with metal centers directly imbedded into the conjugated organic network, which prompts possible interactions between nickel centers and organic ligand fragments in different effective redox states. In most cases, the electrochemical oxidation of nickel–*salen* polymers can be described as a primarily ligand-centered process, which involves the consecutive generation of delocalized radical cations and dications in the conjugated polymer backbone and does not involve the change in the redox state of the metal [11,12,13]. At the same time, nickel ions constitute an essential part of charge transfer pathways in these conducting systems as they mediate the electronic coupling between the two phenolates of each [Ni*salen*] unit. As such, the characteristics of redox states generated in the film during *p*-doping have been shown to depend on the extent of energy match between nickel and *salen* ligand orbitals and successfully modulated through varying electronic and steric properties of ligand substituents [13]. Modification of the metal coordination sphere should also have an effect on the extent of mixing of metal and ligand orbitals in this type of metallopolymers. Such modification could be accomplished for example by the substitution of phenol moieties by isoelectronic aniline groups in Schiff bases.

**Figure 1:**
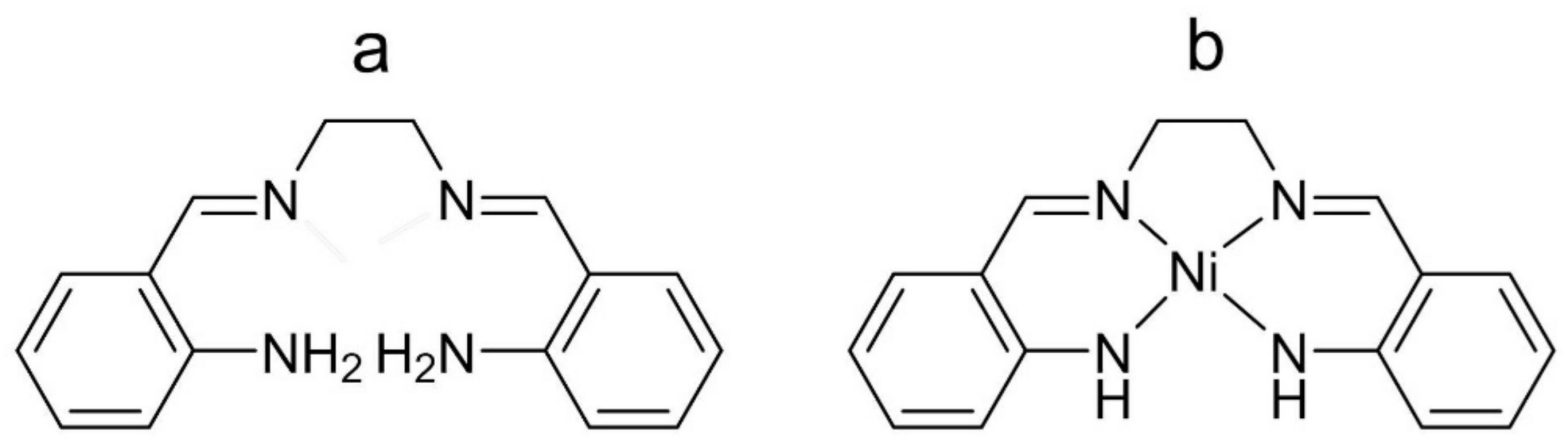
Molecular structures of (**a**) H_2_Amben, and (**b**) its nickel(II) complex [NiAmben].

The N_4_ counterpart of the N_2_O_2_ H_2_Salen ligand, *N*,*N*′-bis(*o*-aminobenzylidene)ethylenediamine, H_2_Amben (Figure 1a), was first described by Pfeiffer et al. in 1937 [14]. Its nickel(II) complex [NiAmben] (Figure 1b) formed via double deprotonation of the ligand in its reaction with a Ni(II) salt belongs to the family of N_4_–anilinosalen complexes comprising two metal-coordinated aminobenzylideneimino moieties connected together through an aliphatic bridge. The studies conducted by several research groups in the 1970s [15,16,17,18] and 1990s [19,20] have shown that [NiAmben] is a square-planar complex that is more easily oxidized than its *salen* counterpart and does not tend to increase its coordination number by the addition of strong nucleophiles, in contrast to its planar four-coordinate N_2_O_2_ analogue. The latter was explained by nearly zero Lewis acidity of the metal and prompted the suggestion that the N_4_ system produces a larger ligand field than the N_2_O_2_ system.

More recent work [21] provided major insights into the electronic structure of the stable radical cations generated by one-electron oxidation of N_4_–anilinosalen complexes with sterically hindered aminobenzylideneimino moieties (i.e., containing substituents in the *ortho* and *para* positions of benzene rings). In particular, it has been shown that they can be described as class III mixed valence compounds according to the Robin Day classification. The anilinyl radicals are delocalized over both aromatic moieties through the nickel ions to the higher extent as compared with the phenoxyl radicals due to better geometric and energetic matches between the anilinyl π-orbitals and out-of-plane nickel 3d orbitals. As a result, the essentially ligand-centered SOMO of the oxidized N_4_–anilinosalen complex acquires substantially higher metallic character than it does in the isoelectronic N_2_O_2_ systems. 

Many nickel–*salen* complexes with free *ortho* and/or *para* positions of phenyl rings tend to undergo oxidative electropolymerization, and similar behavior could be anticipated for their N_4_ analogues. Although previous research studies referred to the oxidation of [NiAmben] as irreversible at high anodic potentials [17], there is only one paper that describes the ability of [NiAmben] to electropolymerize onto a Pt wire under anodic polarization in a 1 M Bu_4_NClO_4_/CH_3_CN solution [22]. The peculiarities of poly-[NiAmben] formation and structure–property relationships in the polymer films with respect to their potential application in energy storing and saving systems have not yet been elucidated.

Herein we report the effect of the Lewis basicity of the solvent on the electrochemical oxidation of [NiAmben] and its ability for oxidative electropolymerization. We investigate functional properties of the generated poly-[NiAmben] films by cyclic voltammetry (CV), electrochemical quartz crystal microbalance (EQCM), in situ UV-Vis spectroelectrochemistry, and in situ conductance measurements to identify the redox states available in the polymer upon *p*-doping. We use the results obtained for poly-[NiSalen] under the same conditions as a comparative model to gain insight into the effect of replacing N_2_O_2_ by N_4_ coordination sphere on the peculiarities of metal–ligand interactions in the system as they define potential-controlled properties of polymeric nickel(II) complexes of Schiff base ligands. 

## 2. Results

### 2.1. Electrochemical Studies of [NiAmben]

The electrochemical behavior of [NiAmben] was investigated by cyclic voltammetry, using three different organic solvents with Et_4_NBF_4_ as the supporting electrolyte. The solvents (DCE, a 50:50 (*v*/*v*) EC/DEC mixture, and DMSO) were selected based on their Gutmann donor numbers (DN) to provide media with varying Lewis basicity (the values of DN are 0 for DCE, 16.4 for EC, 15.1 for DEC, and 29.8 for DMSO). 

**Figure 2:**
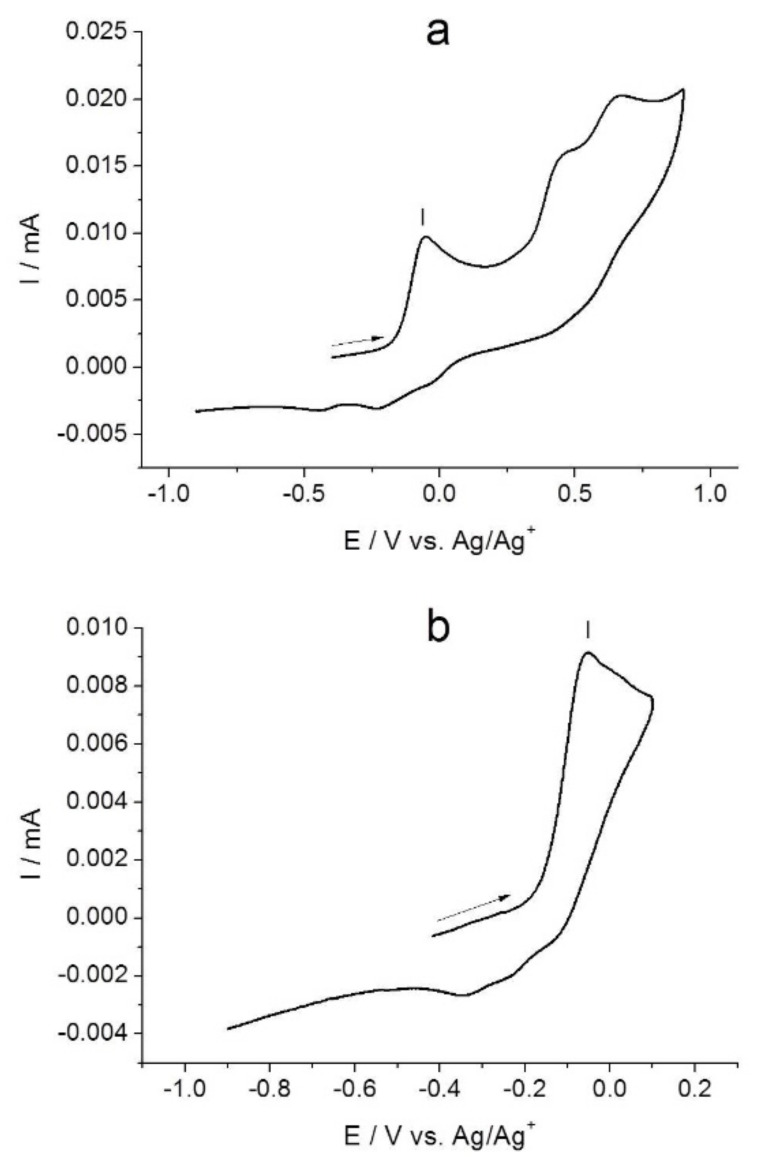
Cyclic voltammograms of a glassy carbon electrode (0.07 cm^2^) in 0.001 mol L^−1^ solution of [NiAmben] in 0.1 M Et_4_NBF_4_/DMSO at a scan rate of 0.05 V s^−1^: (**a**) between −0.9 and 0.9 V; (**b**) between −0.9 and 0.1 V.

The CV curve of [NiAmben] obtained in the range of potentials from −0.9 to 0.9 V vs. Ag/Ag^+^ (all potentials throughout the text are referred to this reference electrode) in the DMSO-based electrolyte displays three well pronounced irreversible oxidation waves with peak potentials at −0.05 V, 0.47 V, and 0.67 V, respectively (Figure 2a). The voltammogram of [NiAmben] recorded in the range of potentials from −0.9 to 0.1 V shows a distinct oxidation peak at the peak potential *E_pa(I)_* = −0.05 V and a poorly defined reduction peak at *E_pc(I)_* = −0.35 V (Figure 2b), which confirms that the first one-electron oxidation of [NiAmben] in DMSO is irreversible.

**Figure 3:**
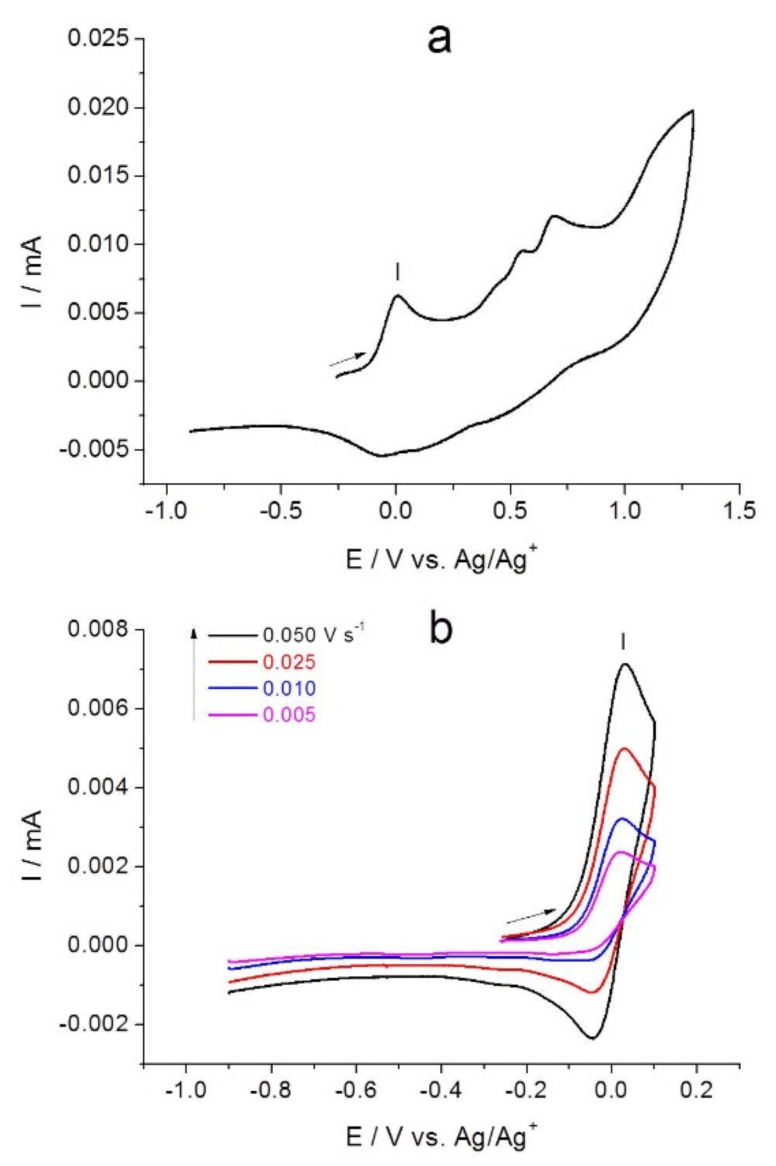
Cyclic voltammograms of a glassy carbon electrode (0.07 cm^2^) in 0.001 mol L^−1^ solution of [NiAmben] in 0.1 M Et_4_NBF_4_/EC/DEC (50:50 *v*/*v*): (**a**) between −0.9 and 1.3 V at a scan rate of 0.05 V s^−1^; (**b**) between −0.9 and 0.1 V at 0.005–0.05 V s^−1^ scan rates.

The cyclic voltammogram of [NiAmben] recorded in the potential range from −0.9 to 1.3 V in the EC/DEC-based electrolyte shows several oxidation waves in the forward scan and a broad reduction wave in the reverse scan (Figure 3a). The first oxidation peak is observed at 0.02 V. The second broad oxidation wave is seen in the range of potentials between ca. 0.3 and 0.9 V (peaks are observed at 0.46 V (sh), 0.55 V, and 0.70 V) and appears to result from a mix of several redox processes at overlapping potentials. The third oxidation event occurs at potentials above 0.9 V but the current does not reach a peak before the direction of the potential sweep is reversed. The CV response of the complex in the range of potentials from −0.9 to 0.1 V (Figure 3b) displays a pair of redox peaks (*E_pa(I)_* = 0.02 V and *E_pc(I)_* = −0.05 V at 0.05 V s^−1^) with the peak potential separation Δ*E* = 70 mV, which is close to the theoretical value for a reversible redox event. The peak current in the forward potential scan (*I_p_*_*forward*_) is higher than that in the return potential scan (*I_p_*_*return*_) at various potential scan rates (0.005–0.05 V s^−1^), and the *I_p_*_*return*_/*I_p_*_*forward*_ relationship gradually decreases upon decreasing the scan rate. This behavior is consistent with low stability of oxidized species and indicates that [NiAmben] undergoes chemically irreversible electron transfer in the EC/DEC mixture in potential range of the first redox event. 

**Figure 4:**
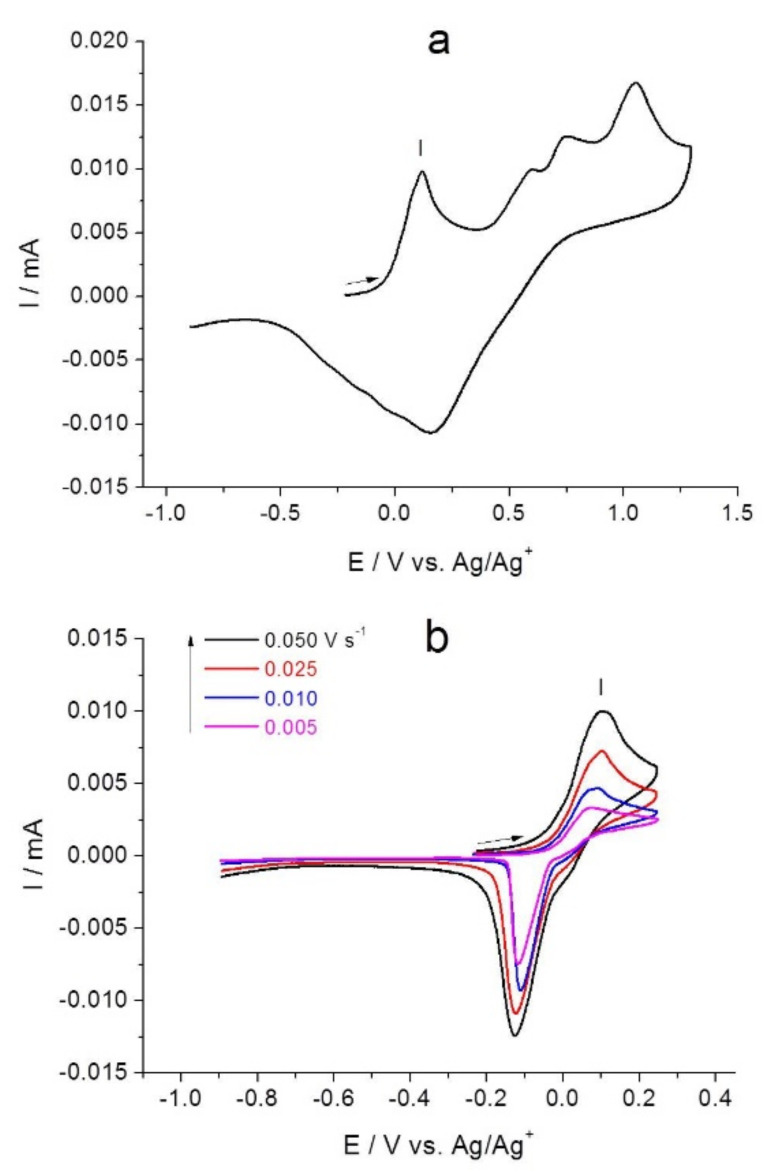
Cyclic voltammograms of a glassy carbon electrode (0.07 cm^2^) in 0.001 mol L^−1^ solution of [NiAmben] in 0.05 M Et_4_NBF_4_/DCE: (**a**) between −0.9 and 1.3 V at a scan rate of 0.05 V s^−1^; (**b**) between −0.9 and 0.25 V at 0.005–0.05 V s^−1^ scan rates.

The CV response of [NiAmben] obtained in the range of potentials between −0.9 and 1.3 V in the DCE-based electrolyte (Figure 4a) shows a well-defined oxidation peak at 0.11 V, followed by two partially overlapping waves at peak potentials of 0.60 V and 0.76 V, and another oxidation peak at 1.05 V. The single reduction wave is broad and better pronounced than the one observed in EC/DEC. The voltammogram of the complex recorded in the potential range from −0.9 to 0.25 V (Figure 4b) displays a pair of well-defined oxidation–reduction peaks (*E_pa(I)_* = 0.11 V, *E_pc(I)_* = −0.13 V at 0.05 V s^−1^) with quite large peak-to-peak separation Δ*E* = 240 mV. The oxidation appears to be chemically reversible. The *I_p_*_*return*_/*I_p_*_*forward*_ relationship does not depend on the scan rate in the range 0.005–0.05 V s^−1^ (Figure 4b), which indicates that the first oxidation of [NiAmben] in DCE could be coupled with reactant adsorption at the electrode. 

The demonstrated irreversibility of one-electron oxidation of [NiAmben] in a DMSO-based electrolyte contrasts to the previously established reversibility of redox processes in [NiSalen] and many other nickel(II) complexes of N_2_O_2_ Schiff base ligands in strong donor solvents (their oxidation yields octahedral Ni(III) species stabilized via axial solvent coordination) [23,24,25]. At the same time, it has been previously shown that the analogue of [NiAmben] that contains two tBu-substituents in the *ortho* and *para* positions of benzene rings is oxidized to a square-planar Ni^II^–anilinyl radical complex regardless of the Lewis basicity of the solvent [21]. The first oxidation of [NiAmben] observed at *E_pa(I)_* could therefore be considered as ligand-based rather than metal-based and ascribed to the formation of Ni^II^–anilinyl radical cation species ([NiAmben]^+•^) in all three solvents. The value of *E_pa(I)_* is shifted to more negative potentials with increasing Gutmann donor number of the electrolyte solvent. Due to its high nucleophilicity, DMSO must be able to react with the formed [NiAmben]^+•^ radical cations [26,27], thus effectively stabilizing them. Such stabilizing interactions between the solvent and the oxidized complex become weaker as the aprotic solvent basicity decreases, rendering the oxidized species less accessible. Repeated cycling in the potential range limited to the first redox event does not affect the voltammetric response of the system in any of the investigated electrolytes.

**Figure 5:**
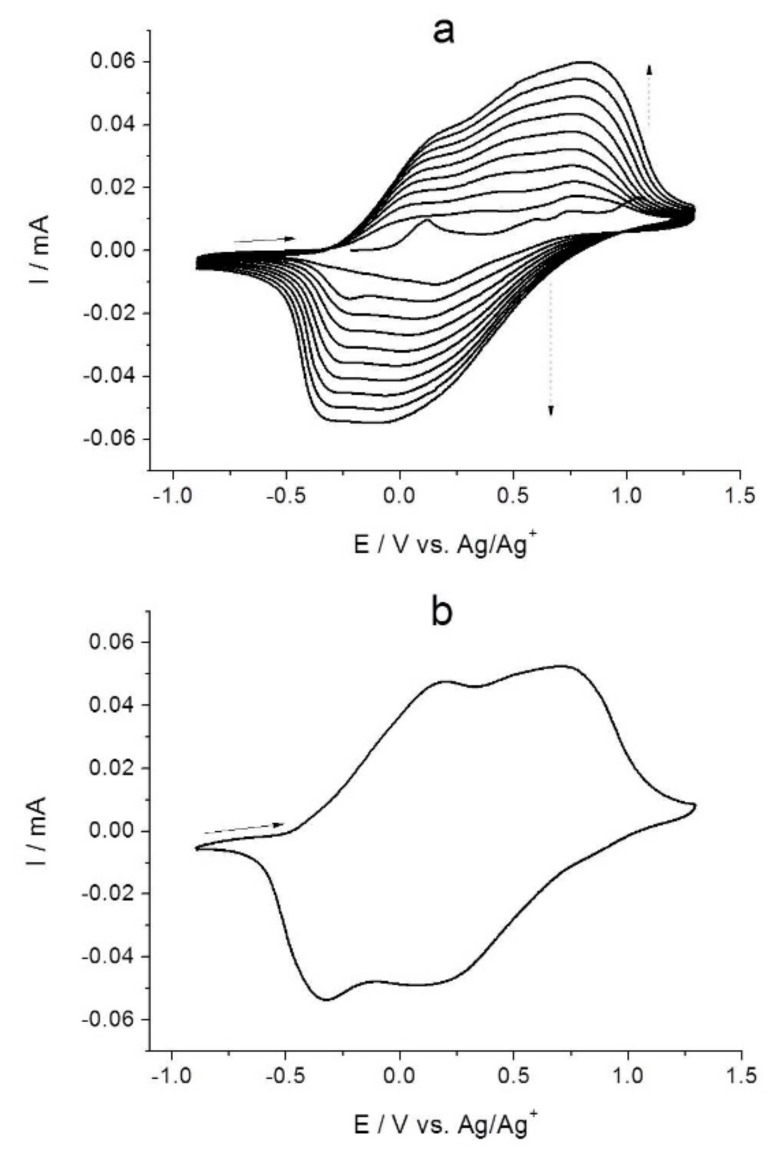
Cyclic voltammograms of a glassy carbon electrode (0.07 cm^2^) in 0.05 M Et_4_NBF_4_/DCE at a scan rate of 0.05 V s^−1^ showing: (**a**) the anodic polymerization of 0.001 mol L^−1^ [NiAmben], and (**b**) the electrochemical response of the poly-[NiAmben] film.

Different numbers, shapes, and positions of redox waves in the voltammograms of [NiAmben] could indicate different reaction pathways for Ni^II^–anilinyl radical cations in different electrolytes as the electrode potential is scanned beyond the potentials of the first oxidation peak. The complexity of CV responses makes it challenging to unambiguously identify the nature of redox processes associated with the observed overlapping redox waves. However, it can be noted that oxidation processes yielding irreversible waves within the potential window from 0.2 to 0.9 V in a DMSO-based electrolyte (Figure 2a) do not induce any significant changes in the voltammetric response of the system upon prolonged electrode potential cycling, so they could be assigned to the subsequent oxidation of DMSO-stabilized [NiAmben]^+•^ species. On the contrary, when the anodic switching potential is set above the onset of the second oxidation in EC/DEC and DCE, the CV peak currents increase with slightly negative shifts in their potentials in successive cycles and new redox waves appear in the voltammetric curves, as compared with the first cycle (Supplementary Materials: Figure S1). Such behavior becomes even more evident when the electrode is subjected to potential cycling in the range between −0.9 and 1.3 V (Figure 5a and Figure S2a). After the electrode is removed from the cell, rinsed with solvent, and transferred to a monomer free-electrolyte solution, its redox response is distinctly different from the one of the bare electrode (Figure 5b and Figure S2b). The results are consistent with irreversible reactions of [NiAmben]^+•^ species yielding an electrochemically active film on the electrode surface. 

The electropolymerization of [NiAmben] proceeds more readily in the solvent of the lowest Lewis basicity, as evidenced by higher polymerization efficiency *Q_redox_*/*Q_poly_* observed in DCE- vs. EC/DEC-based electrolyte (*Q_redox_* is the charge of the redox response of the polymer film, and *Q_poly_* is the total polymerization charge) (Table 1). For each type of electrolyte, the polymerization efficiency of [NiAmben] was found to increase with increasing the anodic switching potential (Table 1). Therefore, the peculiarities of poly-[NiAmben] film formation and redox behavior were further investigated in the DCE-based electrolyte in the potential range between −0.9 and 1.3 V. 

### 2.2. Combined Cyclic Voltammetry and Electrochemical Quartz Crystal Microbalance Characterization of [NiAmben] Polymerization and Redox Processes in Pol-[NiAmben]

**Figure 6:**
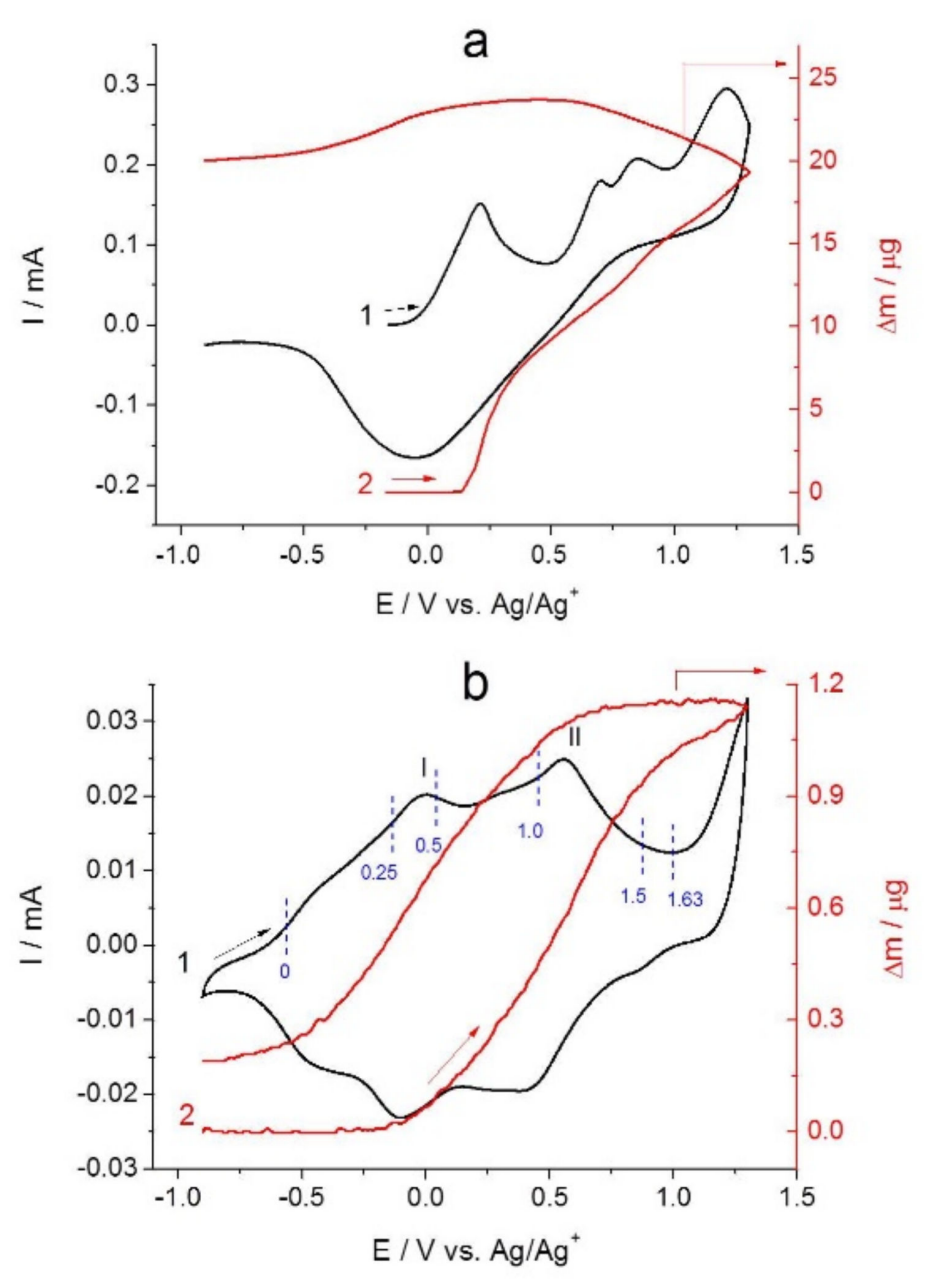
(**a**) Cyclic voltammogram showing electropolymerization of [NiAmben] (0.001 mol L^−1^) at a Pt-coated quartz crystal electrode (1.37 cm^2^) at a scan rate of 0.05 V s^−1^ (black curve 1) and corresponding electrode mass variation with potential (red curve 2); (**b**) CV curve of poly-[NiAmben] at 0.01 V s^−1^ (black curve 1) and corresponding change in electrode mass (red curve 2); blue numbers show oxidation states of the polymer. Electrolyte: 0.05 M Et_4_NBF_4_/DCE.

The oxidative electrochemical deposition of poly-[NiAmben] under potentiodynamic conditions was monitored by the EQCM technique in order to clarify the role of ligand-centered redox reactions of the starting monomer in the polymerization. Cyclic voltammogram of a platinum-coated quartz crystal electrode recorded in the range of potentials from −0.9 to 1.3 V in the DCE-based monomer solution (Figure 6a, curve 1) closely resembles that of the glassy carbon electrode recorded under the same conditions (Figure 4a). As evidenced by curve 2 in Figure 6a, all oxidation processes are accompanied by electrode mass increase, which starts at 0.13 V and continues until 0.38 V in the cathodic half-cycle. The broad reduction wave is accompanied by a decrease in the electrode mass. The observed mass changes are overall consistent with the surface deposition of a positively charged poly-[NiAmben] film doped with BF_4_^−^ ions followed by dedoping of the formed polymer upon reduction and egress of charge-compensating counterions. 

The first oxidation wave with a peak potential at 0.21 V assigned to the formation of Ni^II^–anilinyl radical cation species is accompanied by a sharp increase in the electrode mass, which is however delayed in terms of potential by ca. 230 mV relative to the oxidation onset. The initial fast gain in the electrode mass continues up to ca. 0.3 V and slows down at more anodic potentials. The first oxidation of [NiAmben] is chemically reversible (Figure 4b), so the initial sharp increase in the electrode mass can be attributed to the deposition of polymerization intermediates that can be reversibly reduced if the electrode potential is reversed right after the first oxidation peak (as in the case shown in Figure 4b). If the forward scan is extended beyond the potentials of the first redox process, these adsorbed intermediates undergo further oxidation and continue to react with oxidizing solution species giving rise to anodic waves with peak potentials at 0.70, 0.85, and 1.21 V (Figure 6a) and yielding an insoluble electrochemically active film on the electrode surface. 

**Figure 7:**
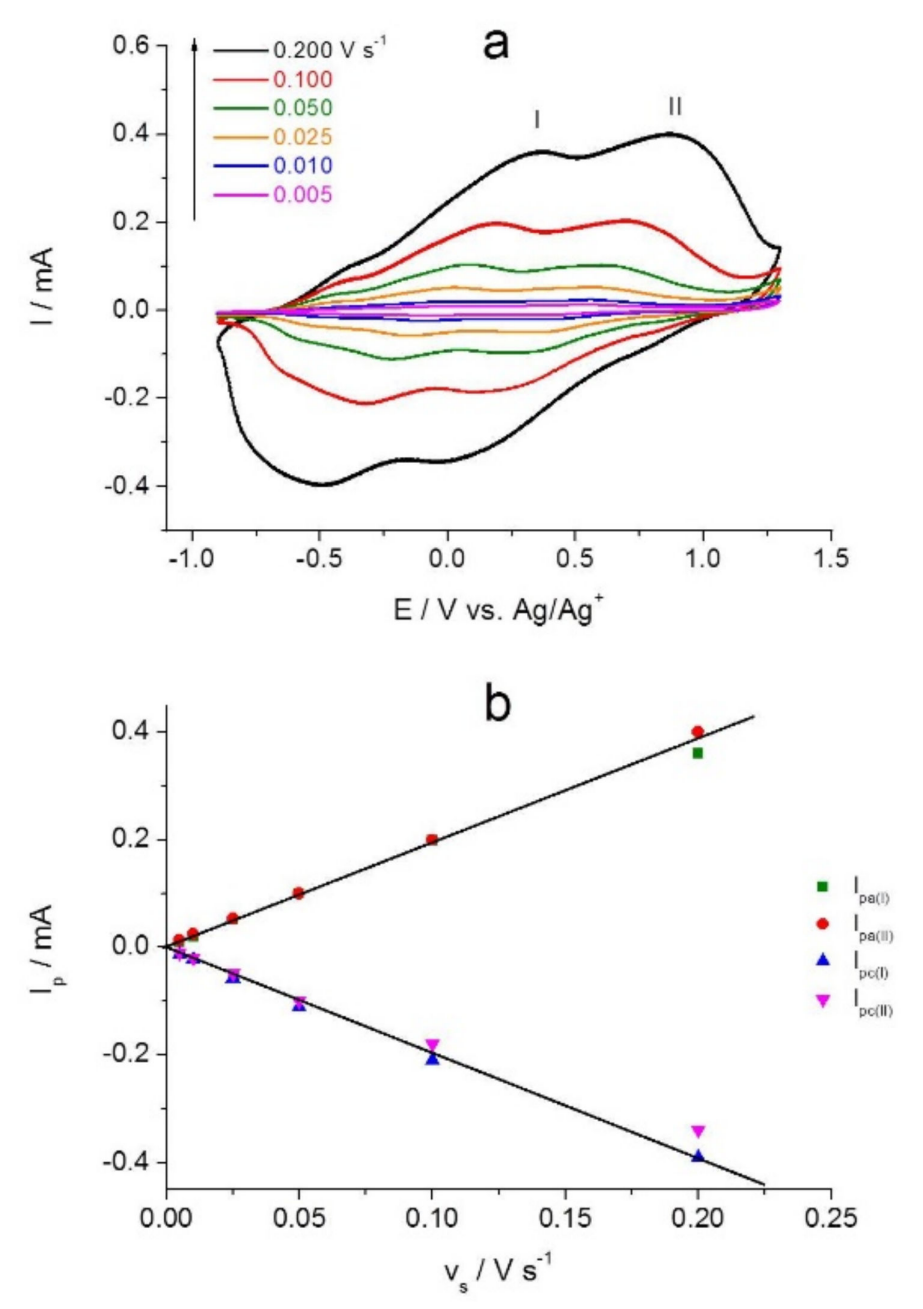
(**a**) CV curves of poly-[NiAmben] (see Figure 6 caption for polymerization conditions) in 0.05 M Et_4_NBF_4_/DCE at 0.005–0.2 V s^−1^ scan rates; (**b**) dependencies of peak currents *I_p_* on the scan rate *v_s_*.

To gain a better understanding of poly-[NiAmben] doping/dedoping and identify the type of electrolyte species that contribute to the redox charge balancing, the electrosynthesized film was further tested by EQCM in a monomer-free electrolyte. Two pairs of oxidation–reduction waves can be clearly observed in CV curves of the polymer-modified electrode (Figure 6b). Their peak potential values *E_pa_*/*E_pc_* are 0.00/−0.10 V and 0.56/0.40 V at a scan rate of 0.01 V s^−1^. Δ*E* increases with increasing the scan rate, *v_s_* (Figure 7a). The anodic and cathodic peak currents are linearly proportional to the scan rate at *v_s_* < 0.1 V s^−1^ (Figure 7b), which points out a thin layer regime of charge transport in the polymer and complete oxidation/reduction of the film in each potential scan. At higher scan rates (>0.1 V s^−1^), the *I_p_* vs. *v_s_* dependence begins to deviate from linearity, which indicates gradual transition from thin layer behavior to the diffusion controlled electrochemical reactions. The electrochemical response of the system remains stable in subsequent cycles until the end of the experiment. 

The coulombic efficiency of poly-[NiAmben] calculated as the ratio of cathodic and anodic voltammetric charges Q_red_/Q_ox_ (Table S1) is close to unity at higher scan rates, which indicates high chemical reversibility of redox transformations in the poly-[NiAmben] film. As the scan rate decreases, so does the Q_red_/Q_ox_ ratio but the value of Q_red_ remains virtually independent of *v_s_*.

The number of electrons exchanged by each [NiAmben] unit of the polymer film in the redox processes, *n*, was calculated using Equation (1) [28]; the results are shown in Table 2.

(1) $$n=\frac{Q_{CV}\cdot M}{F\cdot m_{QCM}}$$

where *Q_CV_* is the voltammetric charge (*Q_ox_* or *Q_red_*), in C; *M* is the molar mass of repeat unit in the undoped polymer, in g mol^−1^; *F* is the Faraday constant (96,485 C mol^−1^); *m_QCM_* is the mass of the dry undoped polymer film determined by microgravimetric measurements, in g. 

As can be seen in Table 2, the dedoping of poly-[NiAmben] is accompanied by reversible transfer of ca. 1.6 electrons per repeat unit in the absence of diffusion limitations (at *v_s_* < 0.1 V s^−1^). Each molecule of the complex possesses several redox-active sites, which justifies the discovered multielectron redox chemistry of the polymer. 

The specific capacity of poly-[NiAmben], *C_mAh_*, can be calculated using the following equation:

(2) $$C_{mAh}=\frac{n\cdot F}{3.6\cdot M}$$

The average specific capacity of poly-[NiAmben] calculated by substituting the value of n = 1.6 into Equation (2) is 136 mAh g^−1^, which is higher than previously found for poly-[NiSalen] and other polymeric nickel(II) complexes of N_2_O_2_ Schiff base ligands [13] and is either on par or exceeding the values of *C_mAh_* for many other redox active polymers reported as perspective materials for electrochemical energy storage devices, e.g., rechargeable batteries [29,30].

The EQCM response accompanying doping/dedoping of the poly-[NiAmben] film shows non-monotonic electrode mass variation Δ*m* with potential (the data obtained at *v_s_* = 0.01 V s^−1^ are shown in Figure 6b as an example). Charge injection, which starts at −0.57 V in the forward scan and continues up to 0.93 V in the reverse scan (Figure S3), is accompanied by an overall increase in the electrode mass. The plot of Δ*m* against the corresponding variation in oxidation charge Δ*Q_ox_* (Figure S4a) shows several reasonably linear regions. The effective molar mass of electrolyte species exchanged during oxidation, *M_ox_*, was calculated from the respective slopes of the plot as described in [31]. The values of *n_ox_* were calculated for each potential range of constant *M_ox_* value by substituting the values of Δ*Q_ox_* into Equation (1). The obtained results are summarized in Table 3. They suggest that charge injection into poly-[NiAmben] proceeds through sequential generation of several redox states in the polymer.

The data presented in Table 3 are also consistent with the overall insertion of electrolyte species into the oxidized film. The calculated values of *M_ox_* are smaller than the mass of BF_4_^−^ ion (87 g mol^−1^) expected to contribute to the compensation of injected charge, which is consistent with the anion and solvent transfer occurring in opposite directions (anion incorporation into the polymer film is accompanied by egress of solvent during oxidation) [32]. The reduction of the polymer shows similar trends of *M_red_* evolution with potential (Figure S4b and Table S2), which are consistent with the egress of charge-compensating ions accompanied by solvent insertion. Values of *M_ox_* and *M_red_* do not remain constant during polymer doping/dedoping, suggesting the varying extent of solvent participation in the electrochemical conversions of poly-[NiAmben] in different redox states. 

### 2.3. In Situ Conductance Studies of Poly-[NiAmben]

**Figure 8:**
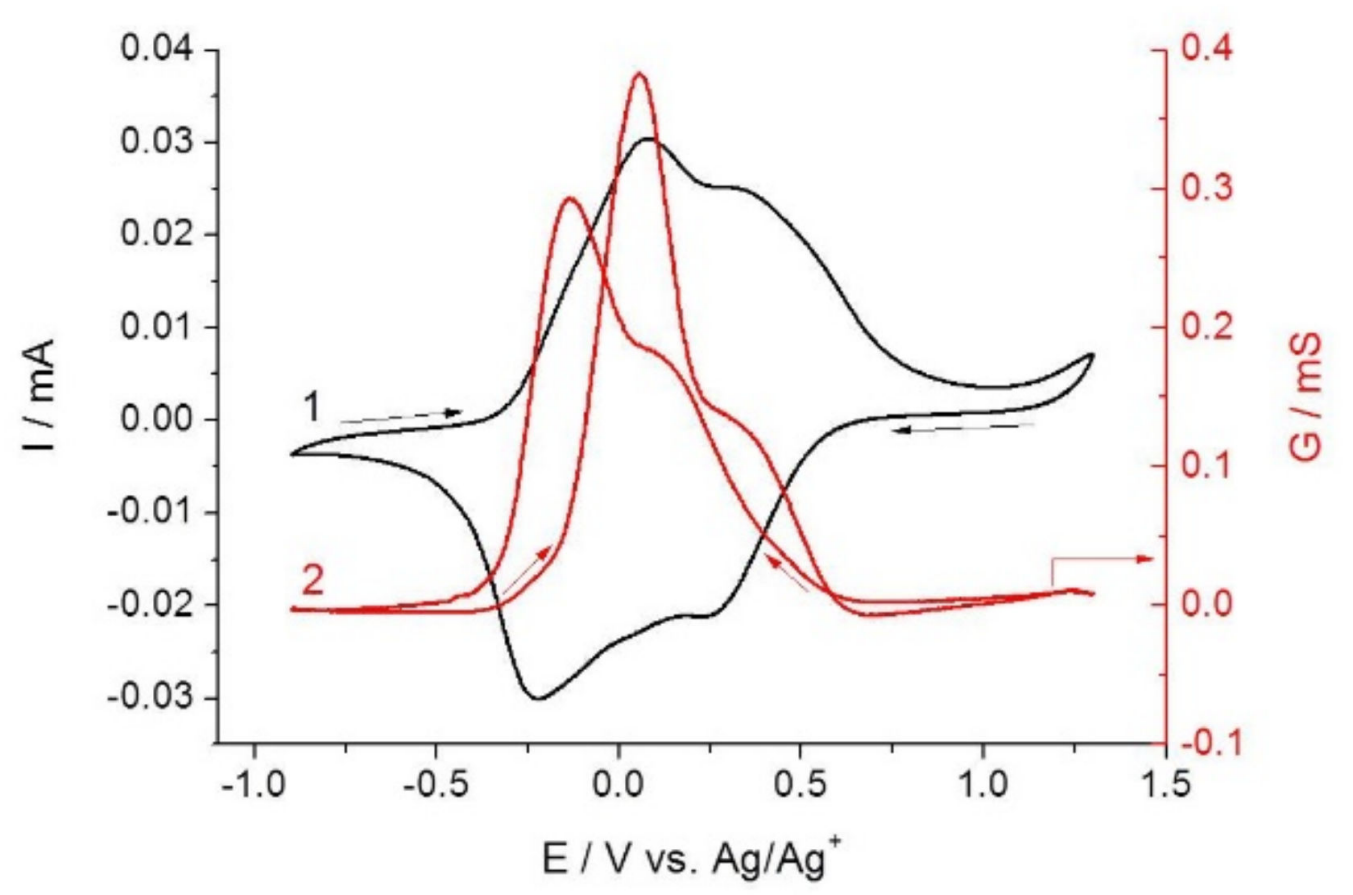
Cyclic voltammogram (black curve 1) and conductance profile (red curve 2) of a poly-[NiAmben]-coated interdigitated electrode in 0.05 M Et_4_NBF_4_/DCE at a scan rate of 0.01 V s^−1^.

In situ conductance measurements were performed on poly-[NiAmben] in order to gain more insight into its electrochemical behavior and related charge transfer phenomena. Reliable values of conductivity cannot be calculated from the experimental data because of inhomogeneity of the polymer film and challenges in the determination of its thickness but the conductivity behavior of the polymer can be indirectly evaluated using its conductance *G* [33]. In this set of experiments, the trends in conductance have been followed as a function of potential. The obtained conductance profile of poly-[NiAmben] and the simultaneously recorded voltammogram are shown in Figure 8. The conduction onset at −0.34 V coincides with the onset for oxidation current. The conductance profile shows a bell-shape with two maxima at 0.04 V and 0.3 V. After dropping to zero at 0.65 V, the polymer conductance immediately starts increasing again but remains nearly an order of magnitude lower than the peak values attained for the less heavily doped film. 

The observed conductivity behavior of poly-[NiAmben] substantially differs from that of conjugated organic polymers, which are usually characterized by a sigmoidal shape conductance profile and conductivity plateau over broad doping potential ranges when polaronic and bipolaronic states co-exist in the *p*-doped films [34,35]. The bell-shaped conductivity indicates that poly-[NiAmben] rather behaves like a redox polymer [36] or a conjugated polymer with a limited number of accessible mixed-valence states [37,38,39,40]. According to the mixed-valence model, the maximum in the bell-shaped conductance profile is reached when half the sites of the redox state are charged [33,41]. Two partially overlapping conductivity regimes are found in poly-[NiAmben] upon charging in the range of potentials from −0.34 V to 0.65 V, which indicates that two mixed valence systems are generated in the polymer film [39,40]. 

### 2.4. In Situ UV-Vis Spectroelectrochemical Studies of Poly-[NiAmben]

Electrochemical oxidation of poly-[NiAmben] from −0.9 V (undoped film) to 1.3 V (heavily *p*-doped film) was closely monitored by in situ UV-Vis spectroscopy to probe different redox states of the polymer and unveil spectral variations with potential indicative of electrochromic properties of the material.

**Figure 9:**
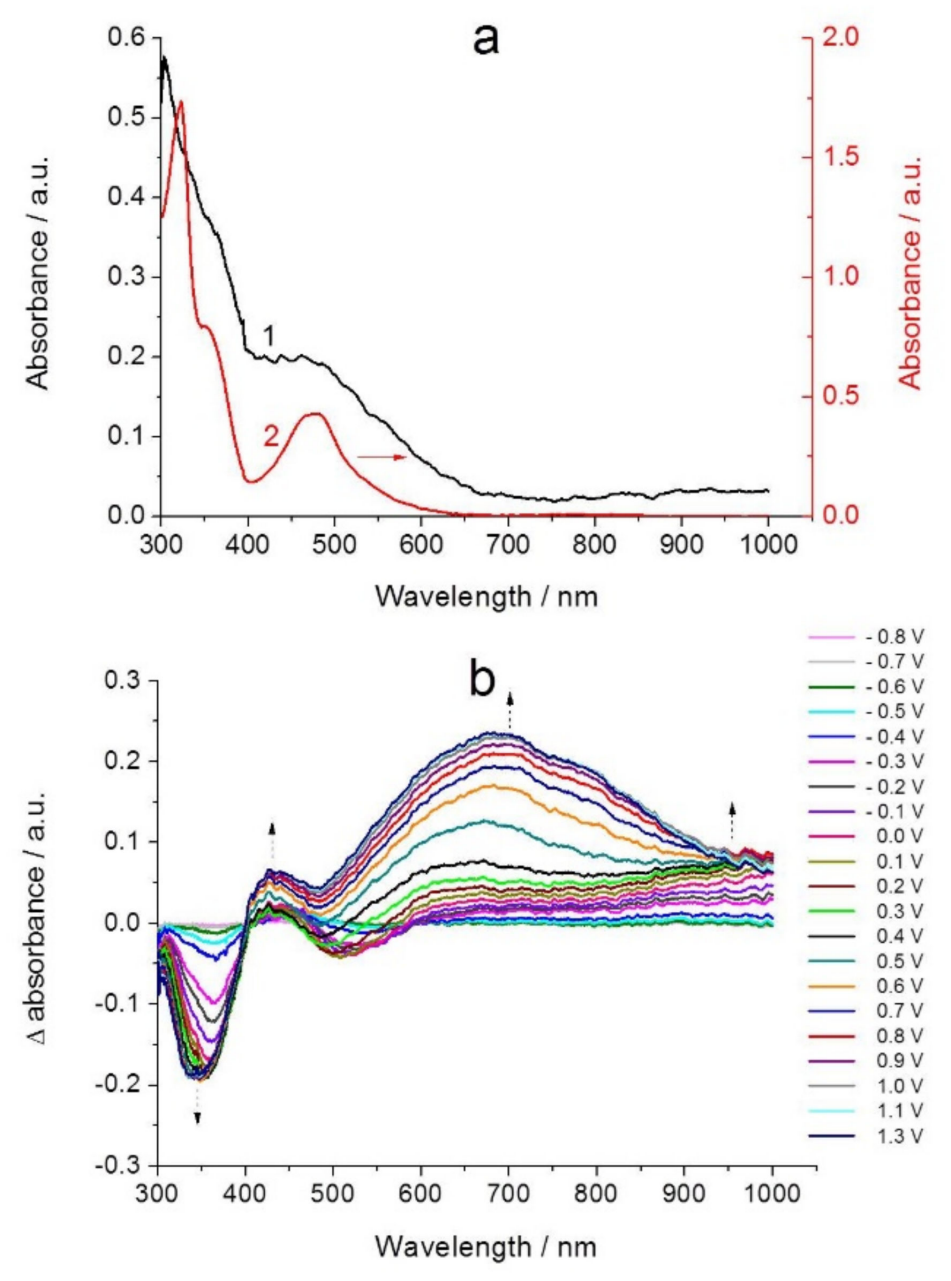
UV-Vis spectra of a poly-[NiAmben]-modified ITO-coated glass electrode in 0.05 M Et_4_NBF_4_/DCE in the potential range from −0.9 to 1.3 V: (**a**) the spectrum of the polymer at −0.9 V (black curve 1) and the spectrum of [NiAmben] monomer (red curve 2); (**b**) differential spectra, referenced to the spectrum of the polymer at −0.9 V.

The spectrum of the undoped poly-[NiAmben] film (collected at −0.9 V) is quite similar to that of [NiAmben] monomer (Figure 9a). In the monomeric complex, the band at 322 nm can be assigned to the intraligand π–π* transition involving the amidobenzilideneimino moieties, the band at 353 nm originates from the CT transition involving delocalized π-orbitals with substantial metal character, and the broad absorption band between 400 and 660 nm with a maximum at 474 nm can be assigned to the nickel-to-amidobenzilideneimino CT transitions involving a metal dz^2^ orbital [21]. Due to the similarity between the polymer and monomer spectra, the same assignment could be made for the bands of poly-[NiAmben] with absorption maxima at 305 nm, 365 nm, and broad absorption centered around ca. 500–550 nm, respectively. 

As the poly-[NiAmben] film is oxidized, the absorption bands of the neutral polymer decrease in intensity while several new bands are formed. Potential-induced spectral changes are presented in Figure 9b, which shows differential spectra of the polymer at step-wise increased potentials referenced to the spectrum of the undoped film (the acquired spectra are shown in Figure S5). 

Electrochemical studies in the range from −0.9 to 1.3 V show that the oxidation of poly-[NiAmben] does not actually begin until the electrode potentials is increased above ca. −0.6 V (Figure S3). As seen from Figure 9b, the π–π* transition of the neutral film at 305 nm is gradually depleted upon polymer oxidation at potentials above −0.6 V. The development of other absorption bands is non-monotonic. Spectral changes below −0.4 V (see also Figure S6a) are minor: the peaks at 330 nm, 360 nm, and 550 nm decrease in intensity while the absorbance between 400 and 500 nm increases in intensity. There is also a slight increase in the absorbance at > 600 nm. These spectral changes accompanied with three isosbestic points at 400 nm, 501 nm, and 592 nm suggest the beginning of conversion between neutral and oxidized moieties of the polymer film. This initial oxidation does not appear to produce any significant number of mobile charges. As the poly-[NiAmben] film is oxidized above −0.4 V (see also Figure S6b), the MLCT band continues decreasing in intensity while undergoing a blue shift. This may be indicative of an increase in the effective oxidation state of the metal due to either metal oxidation [42] or loss of the electron-donating ability of the oxidized aminobenzylideneimino moieties [43]. The bands at 330 nm and 360 nm become further depleted. A new broad absorption band in the NIR range of wavelengths appears in the spectrum (the exact position of the peak cannot be determined because of the spectrophotometer limitations but the increase in absorbance above 900 nm testifies to its existence). Clear isosbestic points at 402 nm, 472 nm, and 576 nm suggest that the polymer conversion from the neutral to the oxidized state continues. Significant increase in the NIR absorption is indicative of efficient charge delocalization. 

With further oxidation above 0 V (see also Figure S6c), a new band at 590 nm appears in the spectrum and starts increasing in intensity while simultaneously undergoing a blue shift (to 550 nm at 0.3 V) and overlapping with the deteriorating MLCT band. The transitions at 330 nm and 360 nm are further depleted. The absorption intensity of the latter band reaches the local minimum at 0.3 V. The absorbance above 900 nm increases. Although the spectral changes are indicative of the continuing oxidation of monomer units, the isosbestic point cannot be clearly identified from the obtained data, suggesting that several electrochemically different chromophores co-exist in the poly-[NiAmben] film in this range of potentials [44].

The changes in the spectra of poly-[NiAmben] in the range of electrode potentials from 0.4 to 0.8 V (see also Figure S6d) are distinctly different from the ones observed at lower anodic potentials. As the absorption band at 330 nm decreases in intensity, the band at 550 nm continues to increase, while NIR absorption remains virtually unchanged. These spectral changes are accompanied by the appearance of new bands at 380 nm, 430 nm, and 690 nm. An additional band is formed at 790 nm at potentials ≥ 0.6 V. The broad bands at 550 nm, 690 nm, and 790 nm largely overlap and increase in intensity as the polymer doping level increases. Only one clear isosbestic point at 347 nm is evident. The absorption band at 330 nm reaches minimum intensity by 0.8 V, indicating that all neutral segments of the metallopolymer have been oxidized. 

Further oxidation of the poly-[NiAmben] film at 0.9 V and 1.0 V (see also Figure S6e) brings up a slight increase in the absorbance at ca. 600 nm and 790 nm while intensities of the band at 380 nm and NIR absorption decrease simultaneously. Two new isosbestic points at 409 nm and 940 nm indicate that the polymer is converted to a heavily doped state. The spectra recorded above 1.0 V do not evolve much, except for a decrease in absorbance at wavelengths >750 nm and a slight increase in the absorption intensity of the band at 600 nm. The observed multistep spectral changes are fully reversible once the electrode potential is reversed.

**Figure 10:**
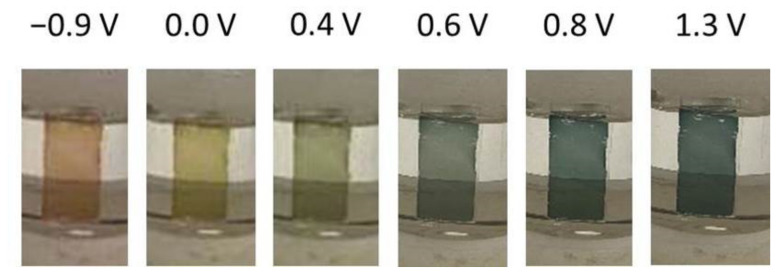
The color change of the poly-[NiAmben] film with varying potentials.

The changes in poly-[NiAmben] spectra accompanying the electrochemical switching of the polymer film between different oxidations states are reflected in visible color changes: the polymer is transformed from the ocher neutral form to the deep blue oxidized form through intermediate redox states displaying yellowish and green colors (Figure 10). The multistep spectral and color changes hold great promise for interesting electrochromic properties of poly-[NiAmben] films. 

## 3. Discussion

The obtained experimental data on the redox processes in [NiAmben] and poly-[NiAmben] films show that they are majorly defined by the specifics of metal–ligand interactions in nickel(II)–N_4_ fragments and can be better understood through a comparison with the thoroughly investigated N_2_O_2_ analogue. 

**Figure 11:**
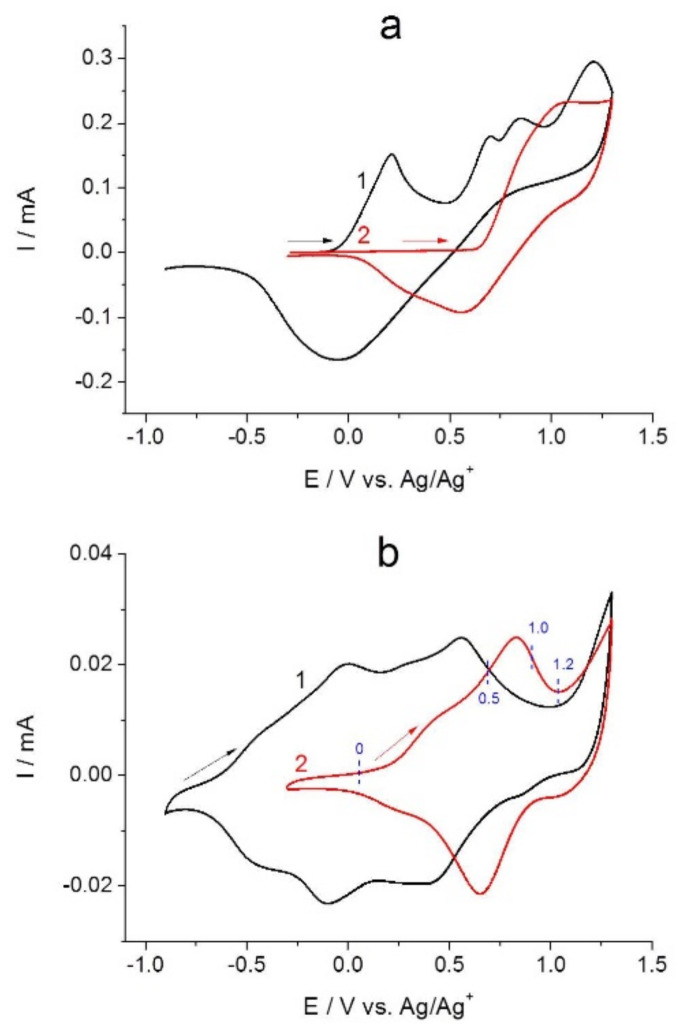
(**a**) Cyclic voltammograms of a Pt-coated quartz crystal electrode (1.37 cm^2^) at a scan rate of 0.05 V s^−1^ in 0.05 M Et_4_NBF_4_/DCE containing 0.001 mol L^−1^ [NiAmben] (black curve 1) or 0.001 mol L^−1^ [NiSalen] (red curve 2); (**b**) CV curves of a Pt-coated quartz crystal electrode (1.37 cm^2^) modified with poly-[NiAmben] (black curve 1) or poly-[NiSalen] (red curve 2) at a scan rate of 0.01 V s^−1^ (blue numbers indicate oxidation states of poly-[NiSalen]).

Figure 11a shows that the onset for the monomer oxidation in a DCE-based electrolyte is significantly cathodically shifted (by ca. 500 mV) in [NiAmben] vs. [NiSalen]. Easier oxidation of the N_4_ complex likely results from higher radical delocalization over ligand scaffold in Ni^II^–anilinyl radical cations [21] as compared with the isoelectronic Ni^II^–phenoxyl radical cation species [45]. 

**Scheme 1:**
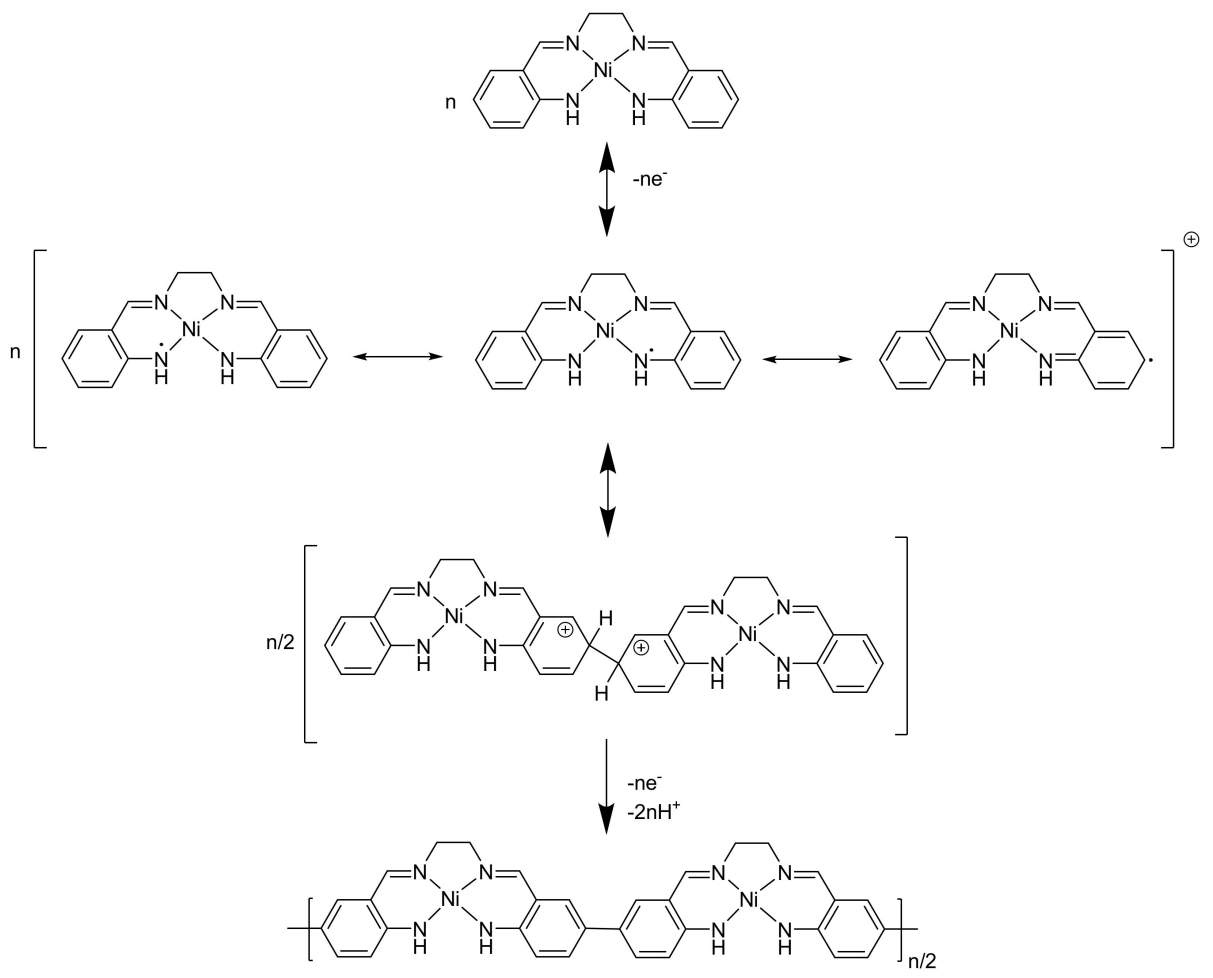
A proposed mechanism for [NiAmben] polymerization.

Generated [NiSalen]^+•^ species are known to undergo radical coupling to yield a redox active metallopolymer, in which phenolate moieties of adjacent monomer units are joined together by carbon–carbon single bonds (this concept has been shared by many researchers [11,12,13] but alternative descriptions of the metal–*salen* polymer structure also exist [46]). Previous studies of one-electron oxidation of the sterically hindered [NiAmben] analogue have shown that the SOMO of generated radicals is more developed on the nitrogens and less on the benzene rings, but the spin density at the phenyl *para* positions is non-negligible [21]. In the absence of *para*-protecting substituents, the electrochemically generated Ni^II^–anilinyl radical cations should be prone to undergo the 4,4′-phenyl–phenyl coupling reaction, yielding an electrochemically active polymer network on the electrode surface. A general representation of the proposed mechanism for [NiAmben] polymerization is shown in Scheme 1. The process likely involves the formation of σ-dimers as intermediate reaction products. Dimeric structures undergo further oxidation, deprotonation, and reactions with other radical cations in the system to yield a robust polymer film. The proposed mechanism of poly-[NiAmben] formation appears to be quite similar to that of poly(diphenylamine) films [47,48] and is indirectly confirmed by the presence of a shoulder at ca. 330 nm in the spectrum of the undoped film (Figure 9a). This shoulder is not observed in the spectrum of the monomer and may result from intraligand π–π* transitions in the σ-bonded amidobenzilideneimino moieties of adjacent [NiAmben] units [39]. 

**Figure 12:**
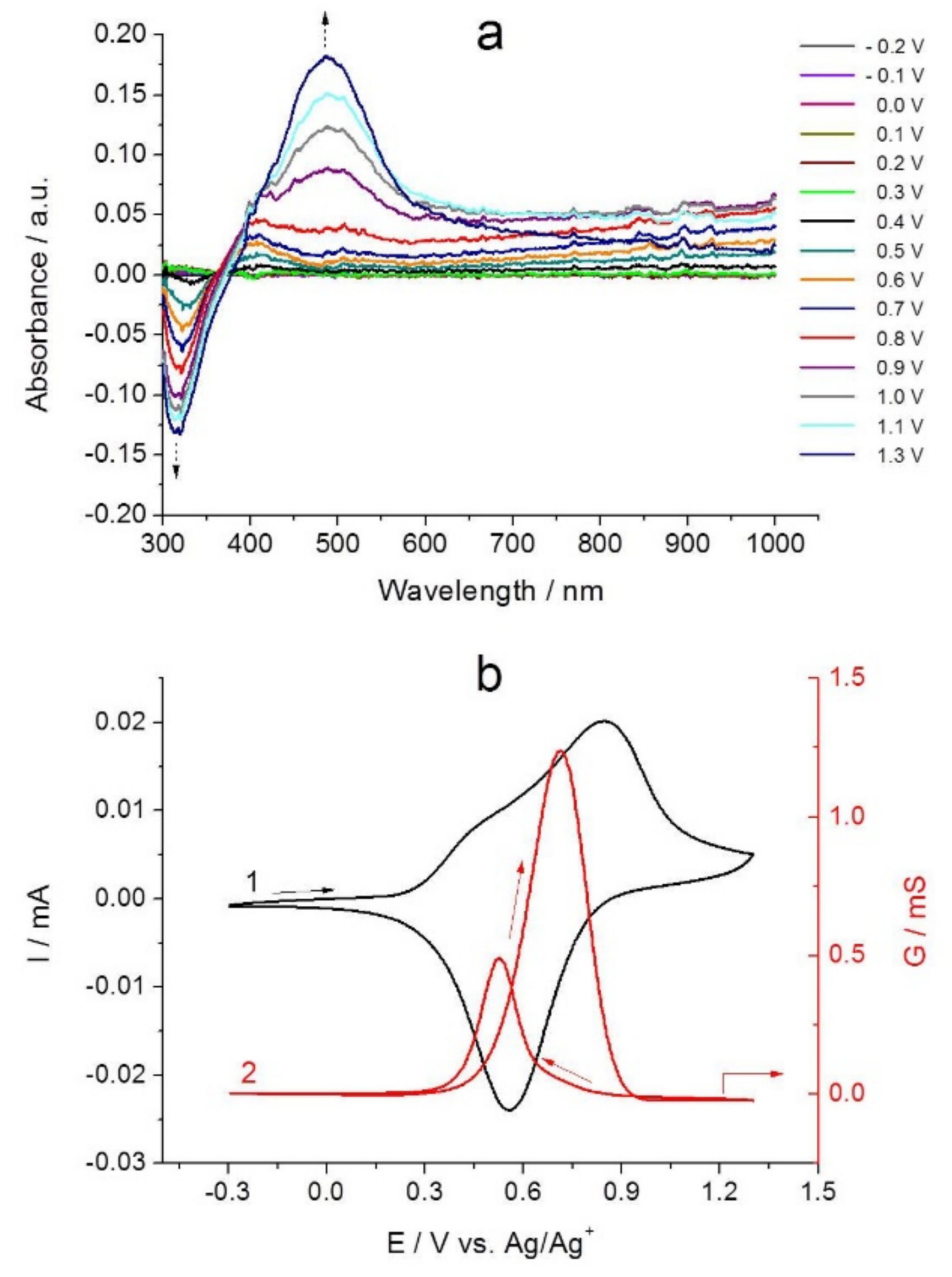
(**a**) Differential UV-Vis spectra of a poly-[NiSalen]-modified ITO-coated glass electrode in 0.05 M Et_4_NBF_4_/DCE in the potential range from −0.9 to 1.3 V, referenced to the spectrum of the polymer at −0.9 V; (**b**) cyclic voltammogram (black curve 1) and conductance profile (red curve 2) of a poly-[NiSalen]-coated interdigitated electrode in 0.05 M Et_4_NBF_4_/DCE at a scan rate of 0.01 V s^−1^.

As follows from Scheme 1, poly-[NiAmben] contains 4,4′-diaminobiphenyl (benzidine) moieties, which are structurally similar to redox-active 4,4′-biphenolate segments in poly-[NiSalen] [11,12,13]. Yet, the analysis of spectral signatures of accessible redox states of the *p*-doped polymer films shows differences in the electronic structure of electrochemically generated charge carriers. The potential-induced changes in the spectra of poly-[NiSalen] in a DCE-based electrolyte observed at potentials below 0.9 V are consistent with the formation of bisphenolic radical cations (according to EQCM studies, this potential range corresponds to the transfer of one electron per monomer unit), and those at more anodic potentials indicate the conversion of radical cations to bisphenolic dications (Figure 12a) [12,13]. 

Spectral changes accompanying the electrooxidation of poly-[NiAmben] are more complex. One-electron oxidation of its repeat units is a two-step process. Potential-induced changes in the spectra of poly-[NiAmben] between −0.6 and 0 V are consistent with formation of anilinyl radical cations that seem to be delocalized through metal centers, as in similar monomeric complexes [21]. Pronounced decrease in the absorption intensity of the MLCT band with increasing electrode potential supports this assumption. As higher anodic charge is injected into the polymer between 0 and 0.4 V, the system gradually shifts to a more stable electronic structure via charge rearrangement between ligand nitrogens and nickel ions. The charged species progressively acquire spectroscopic signatures of benzidine radical cations B^•+^ [39,40,49,50], while involvement of metal orbitals in the oxidation becomes negligible. Potential-induced spectral changes accompanying the transfer of the second electron per a repeat unit of poly-[NiAmben] (above 0.4 V) are overall indicative of the conversion of benzidine radical cations B^•+^ to dications B^2+^) [39,40,49,50]. Spectral changes observed at electrode potentials above 1.0 V are minor, which indicates that poly-[NiAmben]-modified electrodes exhibit capacitive charging rather than faradaic currents in this potential range [51]. 

Different types of charged species in the *p*-doped polymers are responsible for the observed differences in their electrochemical behavior. The generation of anilinyl radical cations delocalized through metal centers in the first step of poly-[NiAmben] oxidation as opposed to the apparent absence of similar charged species in the *p*-doped poly-[NiSalen] film explains significant cathodic shift of the onset potential of the polymer oxidation in the N_4_ system vs. its N_2_O_2_ analogue (Figure 11b). Both polymers feature distinct pairs of redox peaks in the cyclic voltammograms associated with the conversion of the neutral repeat units to singly charged ones (Figure 6b and Figure 11b) and maxima in their conductance profiles at a 1:1 ratio of the neutral/radical cation mixed valence system (Figure 8 and Figure 12b). In addition to that, poly-[NiAmben] shows the second oxidation peak in the CV curve ascribed to the conversion of singly to doubly oxidized fragments and the second conductivity maximum, which is associated with the second redox wave and could therefore be assigned to charge transport through the polymer film involving radical cation/dication mixed valence state. Poly-[NiSalen] undergoes more than one-electron transfer per repeat unit when charged to 1.3 V (Figure 11b), which is not, however, manifested as individual peaks in voltammetric or conductance curves. Instead, this polymer appears to possess negligible conductivity at redox states of n > 1, which indicates suppressed charge mobility between singly and doubly oxidized fragments of the polymer. Previous studies have identified poor intrachain nickel ion-mediated conduction as the main cause of charge trapping in heavily *p*-doped nickel–*salen* polymers [12]. In our tests, poly-[NiSalen] showed decent coulombic efficiencies only at high charge/discharge rates (Figure S8 and Table S3) and quickly degraded during repeated potential cycling when charged to 1.3 V. Enhanced intrachain displacement of charge in the heavily *p*-doped poly-[NiAmben] film prevents the formation of localized immobile charges at high anodic potentials and significantly reduces risks of polymer network overoxidation in multiple charge/discharge cycles. 

Due to improved charge mobility in high oxidation states, poly-[NiAmben] can be reversibly and stably oxidized by the total of ca. 1.6 electrons per repeat unit and deliver high specific capacity of 136 mAh g^−1^, which represents its distinct advantage over the N_2_O_2_ analogue. In addition to that, the increased number of available stable redox states in the polymer provides larger variety of reversible color changes in *p*-doped poly-[NiAmben] as compared with poly-[NiSalen] (Figure S9). Our study thus confirms that tuning metal–ligand interaction through controlled modification of the metal coordination sphere is a powerful strategy for the preparation of metallopolymers that realize improved potential-controlled optical and charge storage properties simultaneously. Such materials could be especially useful for fabricating multifunctional devices that can transform the sustainable energy sector. 

## 4. Materials and Methods

### 4.1. Chemicals and Synthesis

Procedures previously described in the literature were used to synthesize [NiAmben] [15], and [NiSalen] [52]. Their structures were confirmed by ^1^H and ^13^C NMR. Dimethyl sulfoxide (Acros Organics, Fair Lawn, NJ, USA, 99.7%), ethylene carbonate (Acros Organics, 99+%), diethyl carbonate (Sigma-Aldrich, 99%), and 1,2-dichloroethane (Sigma-Aldrich, MO, USA 99.8%) were used as received. Tetraethylammonium tetrafluoroborate Et_4_NBF_4_ (Sigma-Aldrich, 99%) was recrystallized from isopropyl alcohol and dried at 65 °C for 72 h before use. All solutions were prepared in an argon-filled glove box.

### 4.2. Cyclic Voltammetry and EQCM Studies

Electrochemical measurements were performed with a VSP potentiostat (BioLogic Science Instruments, Seyssinet-Pariset, France) at room temperature under an inert atmosphere of argon. A customized single compartment three-electrode electrochemical cell was equipped with a glassy carbon plate as the counter electrode and a non-aqueous Ag/Ag^+^ reference electrode (MW‒1085, BASi) filled with a 0.005 mol L^−1^ AgNO_3_ solution in 0.1 M Et_4_NBF_4_/CH_3_CN (its potential was −0.3 V vs. an Ag/AgCl/sat’d NaCl). 

Cyclic voltammetry experiments were conducted using a glassy carbon disk (MF‒2012, BASi, IN, USA, electrode area 0.07 cm^2^) as the working electrode. The cell was filled with a solution containing a salt/solvent combination (0.05 M Et_4_NBF_4_ in 1,2-dichloroethane (DCE), 0.1 M Et_4_NBF_4_ in a 50:50 (*v*/*v*) mixture of ethylene carbonate (EC) and diethyl carbonate (DEC), or 0.1 M Et_4_NBF_4_ in dimethyl sulfoxide (DMSO)) and 0.001 mol L^−1^ [NiAmben]. Cyclic voltammograms were recorded between −0.9 V and 1.3 V (0.9 V in DMSO) at 0.05 V s^−1^, unless indicated otherwise. When continuous potential cycling in the monomer solution yielded an insoluble deposit on the electrode surface, the electrode was transferred to the monomer-free solution, and cyclic voltammograms were recorded between −0.9 V and 1.3 V at 0.05 V s^−1^.

For the combined CV/EQCM characterization tests of poly-[NiAmben] and poly-[NiSalen], a VSP potentiostat was connected to a 5 MHz QCM100 Quartz Crystal Microbalance equipped with a Metex MXC 1600 frequency counter (Metex Co, Seoul, Korea). A platinum-coated quartz crystal (electrode area 1.37 cm^2^) was used as the working electrode. The oscillation frequency of the crystal was measured by QCM in argon before the immersion in the solution for the polymerization. Polymer films were deposited onto the working electrode from a 0.05 M Et_4_NBF_4_/DCE solution containing 0.001 mol L^−1^ monomer by a single potential scan between −0.9 V (−0.3 V for [NiSalen]) and 1.3 V at a 0.05 V s^−1^ scan rate. Before being subjected to characterization tests, the polymer-modified crystal was dried in argon until it reached a constant oscillation frequency (for about 30 min). The mass of the dry polymer film, *m_QCM_*, was then calculated by using the Sauerbrey equation [53], which relates the mass change per unit area at the electrode surface to the observed change in the oscillation frequency of the crystal. We found *m_QCM_* = 5.3 µg for poly-[NiAmben] and *m_QCM_* = 3.5 µg for poly-[NiSalen], respectively. The CV curves of the polymer films were obtained between −0.9 V (−0.3 V for poly-[NiSalen]) and 1.3 V at 0.005, 0.01, 0.025, 0.05, 0.1, and 0.2 V s^−1^ scan rates. At least three consecutive cycles were recorded at each scan rate.

### 4.3. In Situ Conductance Measurements

In situ conductance measurements were carried out in a bipotentiostat regime with the same electrochemical cell as was used for cyclic voltammetry. Interdigitated Pt electrodes (MicruX Technologies, Gijón, Spain) with the comb distance of 5 µm served as the working electrodes. The polymer films were deposited through a CV mode at a scan rate of 0.01 V s^−1^. A constant bias of 5 mV was applied between the combs of the interdigitated electrode and the flowing current was measured as a function of potential. The conductance of the polymer was calculated from the measured current between the combs by applying Ohm’s law [33]. The polymer deposition process was intentionally terminated when the conductance reached a plateau, which indicated that the polymer completely filled the gap. The electrodes were then transferred to a monomer-free solution wherein in situ conductance measurements of the polymer films at 10 mV bias were performed in parallel to the voltammetric measurements at 0.01 V s^−1^.

### 4.4. UV-Vis Spectroscopy and In Situ UV-Vis Spectroelectrochemical Studies

UV-Vis absorption spectra of [NiAmben] and [NiSalen] (1 × 10^-4^ M, DCE) were collected in 10 mm pathlength quartz cuvettes using an SF‒2000 spectrophotometer (OKB Spectr, Saint Petersburg, Russia). The spectrophotometer was combined with a VSP potentiostat for in situ UV-Vis spectroelectrochemical investigation of the polymer films. The working electrode was an indium tin oxide (ITO)-coated conducting glass (Sigma Aldrich, electrode area 1.0 cm^2^). A Pt wire was used as the counter electrode and an AgCl-coated Ag wire was used as the pseudo-reference electrode (its potential was close to that of an Ag/AgCl/sat’d NaCl). Electrodes were placed in a custom-made three-electrode quartz cell. Spectra were acquired in the range 300–1000 nm at fixed potentials incrementally stepped in 0.1 V intervals from −0.9 V (−0.3 V for poly-[NiSalen]) to 1.3 V (the spectra at 1.2 V have not been registered to avoid possible overoxidation of the polymers). All spectra were collected under stationary conditions (after the current remained unchanged for 120 s). 

## 5. Conclusions

The present study demonstrates that a nickel(II) N_4_–anilinosalen complex [NiAmben] is irreversibly oxidized in aprotic solvents and undergoes polymerization in solvent media of low and medium Lewis basicity. The highest polymerization efficiency is observed in 1,2-dichloroethane, wherein [NiAmben] appears to undergo carbon–carbon coupling in the *para* positions of aromatic rings, yielding a robust polymer structure. The resulting poly-[NiAmben] film is electrochemically active and electrically conductive within a wide range of electrode potentials, showing two well-defined pairs of voltammetric peaks and two conductivity maxima, which reflects the switching of repeat units of the polymer between neutral, radical cationic, and dicationic states. The metallopolymer shows reversible color changes between ocher, yellow, green, and deep blue, demonstrating the electrochromic behavior concurrently with the redox reactions. Poly-[NiAmben] clearly possesses enhanced functional properties over its N_2_O_2_ counterpart, poly-[NiSalen]. The advantages are largely due to the increased number of available stable redox states in the polymer. High extent of mixing of nickel(II) and ligand orbitals affords efficient charge rearrangement between redox noninnocent ligand moieties and nickel ions, and hence efficient intrachain delocalization of generated charges, contributing to high coulombic efficiency, conductivity, and stability of poly-[NiAmben], even in the heavily doped state. Impressive potential-controlled optical and charge storage properties open up multiple possibilities of using this polymer as electrode material in energy storage and saving applications.

## Figures and Tables

**Table 1:** The efficiency of [NiAmben] electropolymerization (*Q_redox_*/*Q_poly_*) at a glassy carbon electrode (0.07 cm^2^) in different electrolytes and potential ranges at a 0.05 V s^−1^ scan rate.

Electrolyte Solvent	Potential Range, V	*Q_redox_*/*Q_poly_*
EC/DEC	−0.9 to 0.5	0.03
	−0.9 to 1.3	0.18
DCE	−0.9 to 0.65	0.22
	−0.9 to 1.3	0.55

**Table 2:** Oxidation state of poly-[NiAmben] during voltammetric oxidation/reduction in 0.05 M Et_4_NBF_4_/DCE.

Scan Rate, *v_s_* (V s^−1^)	Electrons Exchanged Per Repeat Unit (Oxidation), *n_ox_*	Electrons Exchanged Per Repeat Unit (Reduction), *n_red_*
0.005	2.35	1.63
0.010	2.09	1.62
0.025	1.85	1.62
0.050	1.78	1.59
0.100	1.68	1.57
0.200	1.60	1.53

**Table 3:** Effective molar mass of electrolyte species, *M_ox_*, and the number of electrons per repeat unit, *n*, sequentially exchanged during voltammetric oxidation of poly-[NiAmben] in 0.05 M Et_4_NBF_4_/DCE at 0.01 V s^−1^ scan rate.

Potential Range, V	Effective Molar Mass of Electrolyte Species, *M_ox_* (g mol^−1^)	Electrons Exchanged Per Repeat Unit, *n_ox_*
−0.57 to −0.16	3	0.25
−0.16 to 0.05	18	0.25
0.05 to 0.45	42	0.5
0.45 to 1.00	59	0.63
1.00 to 0.93 (reverse scan)	17	0.46

## Data Availability

The data presented in this study are available on request from the corresponding author.

## Supplementary Materials

The following are available online, Figure S1: Cyclic voltammograms (50 consecutive cycles) of a glassy carbon electrode (0.07 cm^2^) at a scan rate of 0.05 V s^−1^ in 0.001 mol L^−1^ solution of [NiAmben]: (**a**) in 0.1 M Et_4_NBF_4_/EC/DEC (50:50 *v*/*v*) between −0.9 and 0.5 V; (**b**) in 0.05 M Et_4_NBF_4_/DCE between −0.9 and 0.65 V. The 1st and 50th cycles are shown, Figure S2: Cyclic voltammograms of a glassy carbon electrode (0.07 cm^2^) in 0.1 M Et_4_NBF_4_/EC/DEC (50:50 *v*/*v*) between −0.9 and 1.3 V at a scan rate of 0.05 V s^−1^ showing: (**a**) the anodic polymerization of 0.001 mol L^−1^ [NiAmben]; (**b**) the electrochemical response of the resulting poly-[NiAmben] film, Table S1: Coulombic efficiency of poly-[NiAmben] in the potential range between −0.9 and 1.3 V in 0.05 M Et_4_NBF_4_/DCE, Figure S3: Dependence of the redox charge Δ*Q* on the electrode potential *E* for the CV curve of poly-[NiAmben] shown in Figure 6b, Figure S4: Mass–charge plots for the EQCM test of poly-[NiAmben] shown in Figure 6b: (**a**) polymer oxidation; (**b**) polymer reduction, Table S2: Effective molar mass of electrolyte species, *M_red_*, and the number of electrons per repeat unit, n, exchanged during voltammetric reduction of poly-[NiAmben] in 0.05 M Et_4_NBF_4_/DCE at 0.01 V s^−1^ scan rate, Figure S5: UV-Vis spectra of a poly-[NiAmben]-modified ITO-coated glass electrode collected in 0.05 M Et_4_NBF_4_/DCE in the potential range from −0.9 to 1.3 V, Figure S6: Differential UV-Vis spectra of a poly-[NiAmben]-modified ITO-coated glass electrode during redox switching in 0.05 M Et_4_NBF_4_/DCE: (**a**) spectra from −0.8 to −0.4 V, referenced to the spectrum of the polymer at −0.9 V; (**b**) spectra from −0.3 to 0.0 V, referenced to the spectrum of the polymer at −0.4 V; (**c**) spectra from 0.1 to 0.3 V, referenced to the spectrum of the polymer at 0.0 V; (**d**) spectra from 0.4 to 0.8 V, referenced to the spectrum of the polymer at 0.3 V; (**e**) spectra from 0.9 to 1.3 V, referenced to the spectrum of the polymer at 0.8 V, Figure S7: UV-Vis spectra of a poly-[NiSalen]-modified ITO-coated glass electrode at −0.3 V acquired in 0.05 M Et_4_NBF_4_/DCE (black curve 1) and the spectrum of [NiSalen] monomer (red curve 2), Figure S8: CV curves of a poly-[NiSalen]-modified Pt-coated quartz crystal electrode (1.37 cm^2^) in 0.05 M Et_4_NBF_4_/DCE between −0.3 and 1.3 V at different scan rates (0.005–0.2 V s^−1^), Table S3: Coulombic efficiency of poly-[NiSalen] in the potential range between −0.3 and 1.3 V in 0.05 M Et_4_NBF_4_/DCE at different scan rates (calculated from the CV data shown in Figure S8), Figure S9: The color change of the poly-[NiSalen] film with varying potentials.

## Sample Availability

Samples of the compounds are available on request from the corresponding author.
