# Supplementary material for: Nickel(II) Complex of N4 Schiff Base Ligand as a Building Block for a Conducting Metallopolymer with Multiple Redox States
Source: Molecules. 2021 Apr 30;26(9):2646. doi: 10.3390/molecules26092646 (PMC8124806; doi:10.3390/molecules26092646)
Supplement: Supplementary file 1 [file molecules-26-02646-s001.zip › molecules-1194555-supplementary.pdf]

Supplementary Materials

Nickel(II) Complex of N_4 Schiff Base Ligand as a Building Block for a Conducting Metallopolymer with Multiple Redox States

Mikhail Karushev*, Evgenia Smirnova, Irina Chepurnaya

Ioffe Physical-Technical Institute of the Russian Academy of Sciences (Ioffe Institute),

26 Polytechnicheskaya str., St. Petersburg, 194021, Russia

*corresponding author. e-mail: mkarushev@mail.ioffe.ru

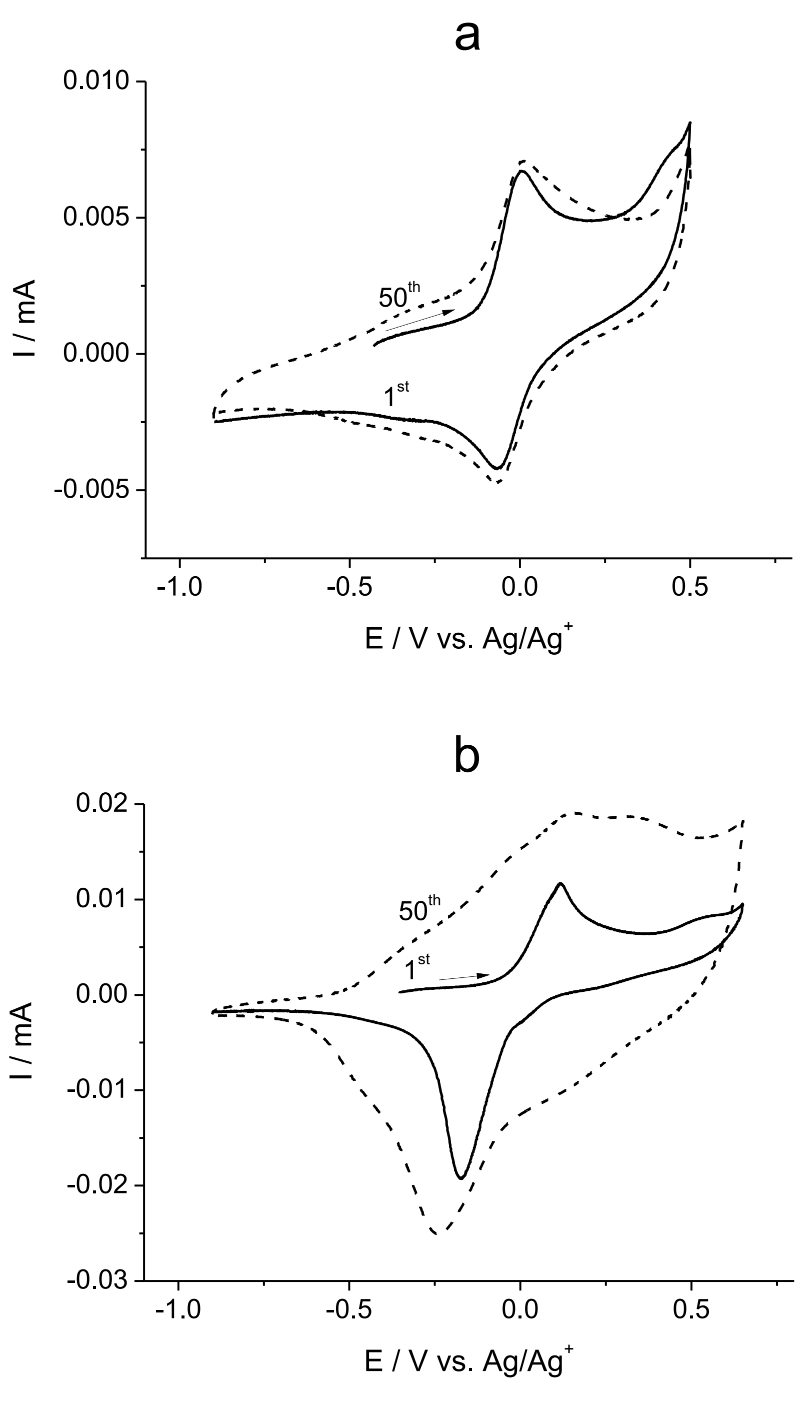

Figure S1. Cyclic voltammograms (50 consecutive cycles) of a glassy carbon electrode (0.07 cm^2) at a scan rate of 0.05 V s^{-1} in 0.001 mol L^{-1} solution of [NiAmben]: (**a**) in 0.1 M Et_4NBF_4/EC/DEC (50:50 v/v) between −0.9 and 0.5 V; (**b**) in 0.05 M Et_4NBF_4/DCE between −0.9 and 0.65 V. The 1st and 50th cycles are shown.

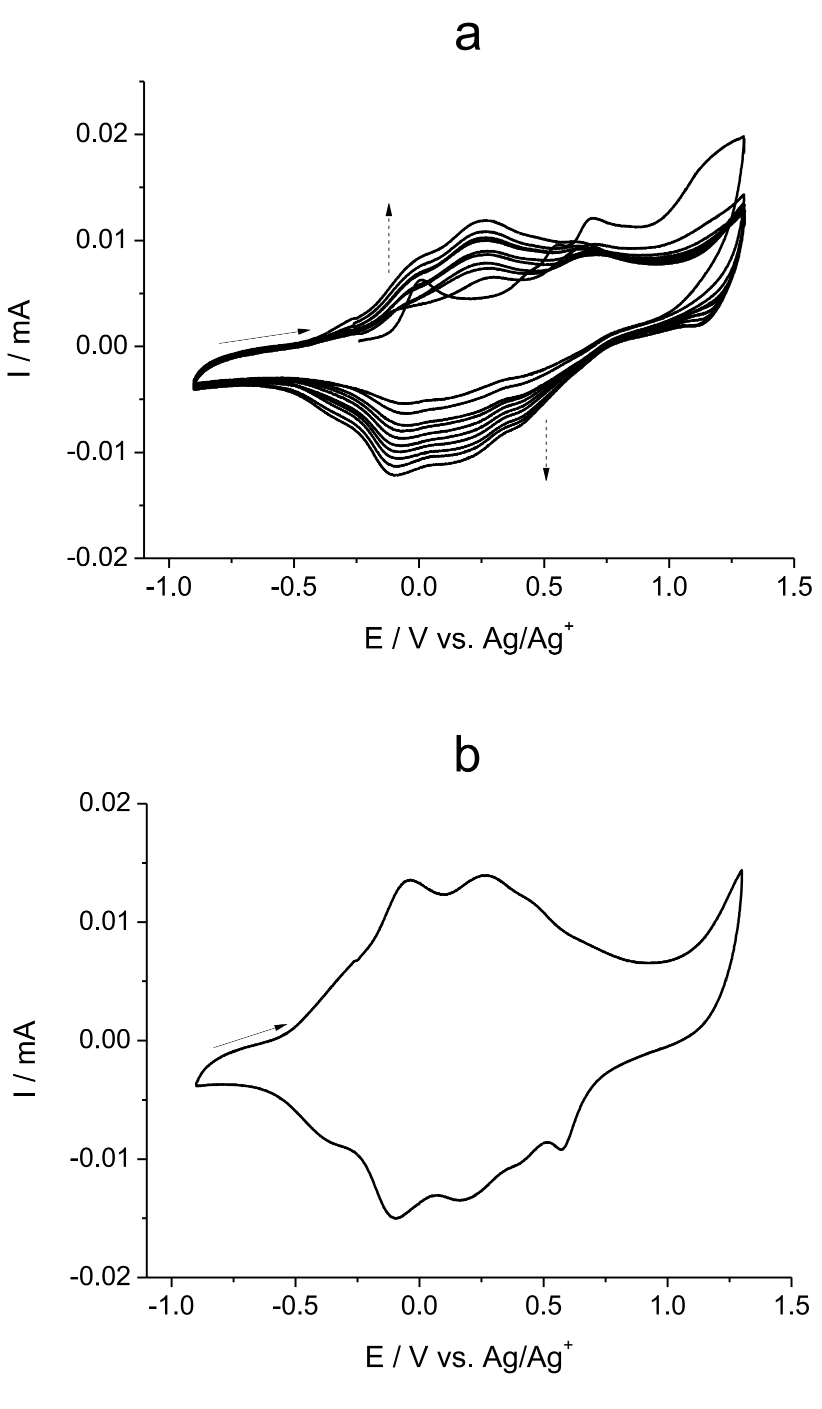

Figure S2. Cyclic voltammograms of a glassy carbon electrode (0.07 cm^2) in 0.1 M Et_4NBF_4/EC/DEC (50:50 v/v) between −0.9 and 1.3 V at a scan rate of 0.05 V s^{-1} showing: (**a**) the anodic polymerization of 0.001 mol L^{-1} [NiAmben]; (**b**) the electrochemical response of the resulting poly-[NiAmben] film.

Table S1. Coulombic efficiency of poly-[NiAmben] in the potential range between −0.9 and 1.3 V in 0.05 M Et_4NBF_4/DCE.

Scan rate, v_s (V s^{-1})	Injected (oxidation) charge, Q_{ox} (mC)	Recovered (reduction) charge, Q_{red} (mC)	Coulombic efficiency, Q_{red} / Q_{ox}
0.005	3.74	2.60	0.70
0.01	3.33	2.58	0.77
0.025	2.95	2.58	0.87
0.05	2.83	2.54	0.90
0.1	2.67	2.50	0.94
0.2	2.55	2.43	0.95

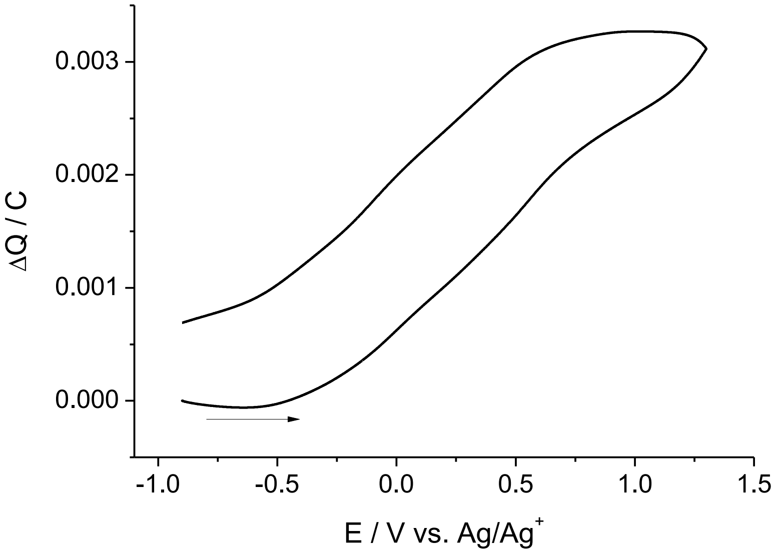

Figure S3. Dependence of the redox charge ΔQ on the electrode potential E for the CV curve of poly-[NiAmben] shown in Figure 6b.

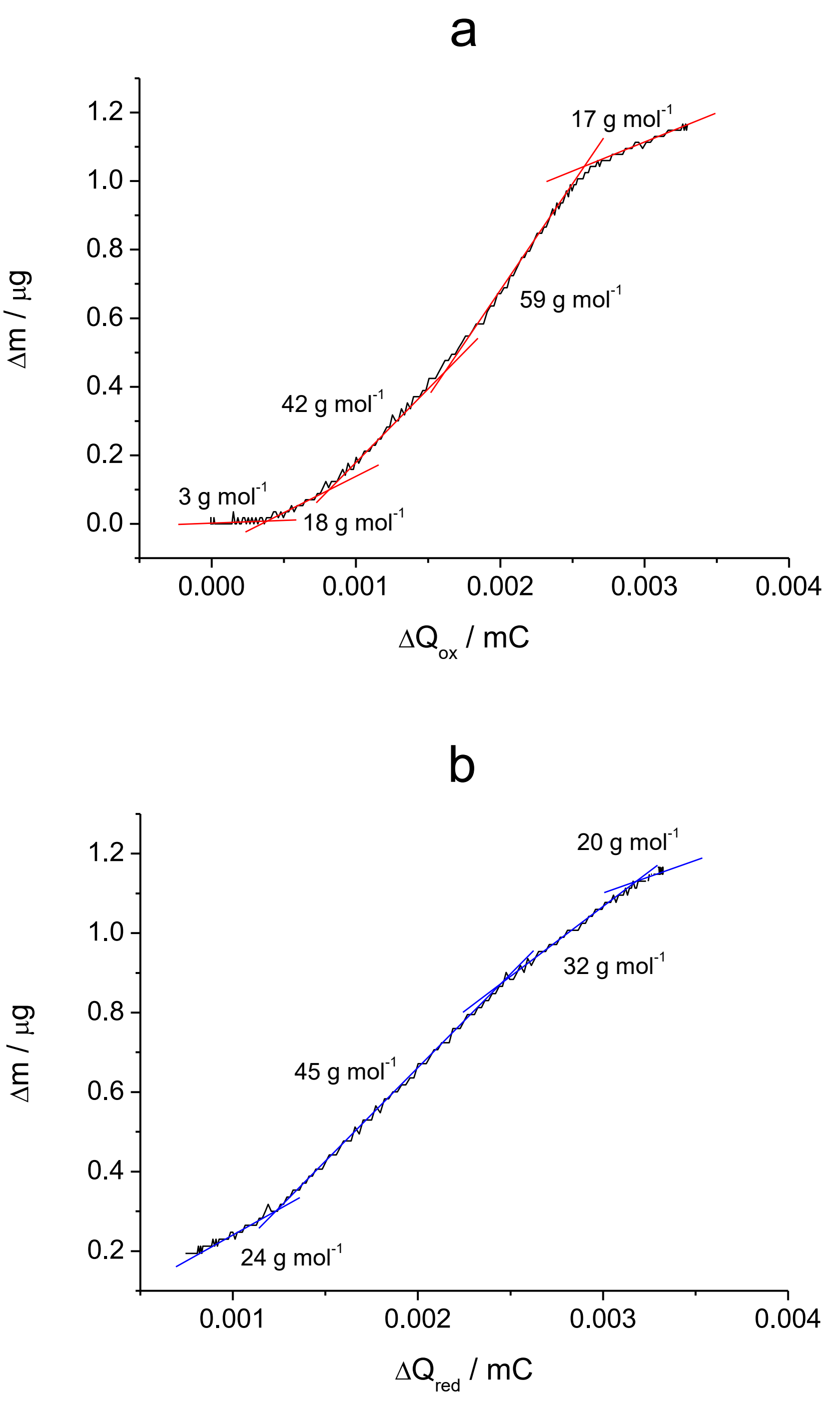

Figure S4. Mass-charge plots for the EQCM test of poly-[NiAmben] shown in Figure 6b: (**a**) polymer oxidation; (**b**) polymer reduction.

Table S2. Effective molar mass of electrolyte species, M_{red}, and the number of electrons per repeat unit, n, exchanged during voltammetric reduction of poly-[NiAmben] in 0.05 M Et_4NBF_4/DCE at 0.01 V s^{-1} scan rate.

Potential range, V	Effective molar mass of electrolyte species, M_{red} (g mol^{-1})	Electrons exchanged per repeat unit, n_{red}
0.93 to 0.66	20	0.08
0.66 to 0.24	32	0.43
0.24 to −0.41	45	0.81
−0.41 to −0.9	24	0.3

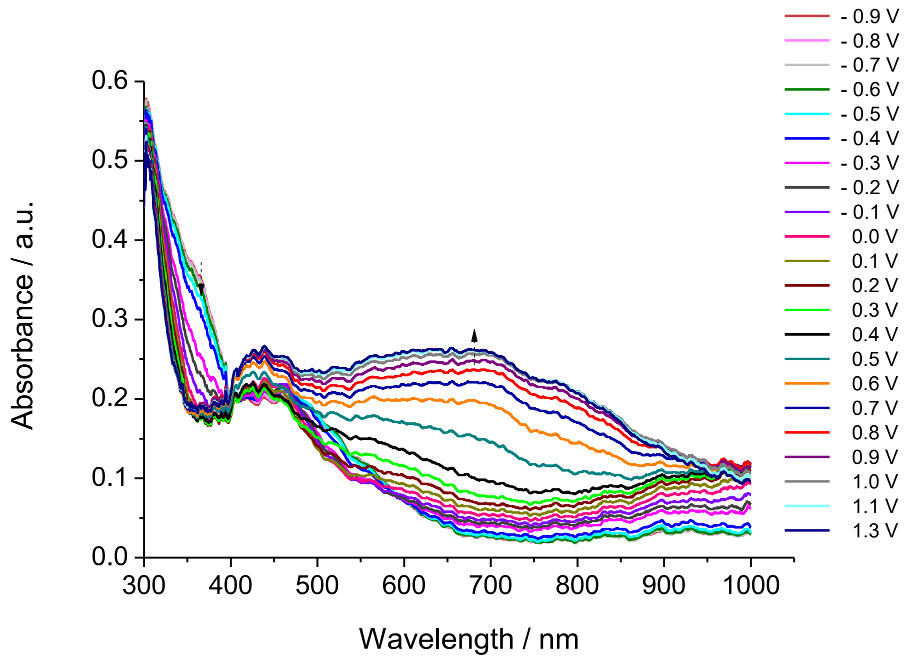

Figure S5. UV-Vis spectra of a poly-[NiAmben]-modified ITO-coated glass electrode collected in 0.05 M Et_4NBF_4/DCE in the potential range from −0.9 to 1.3 V.

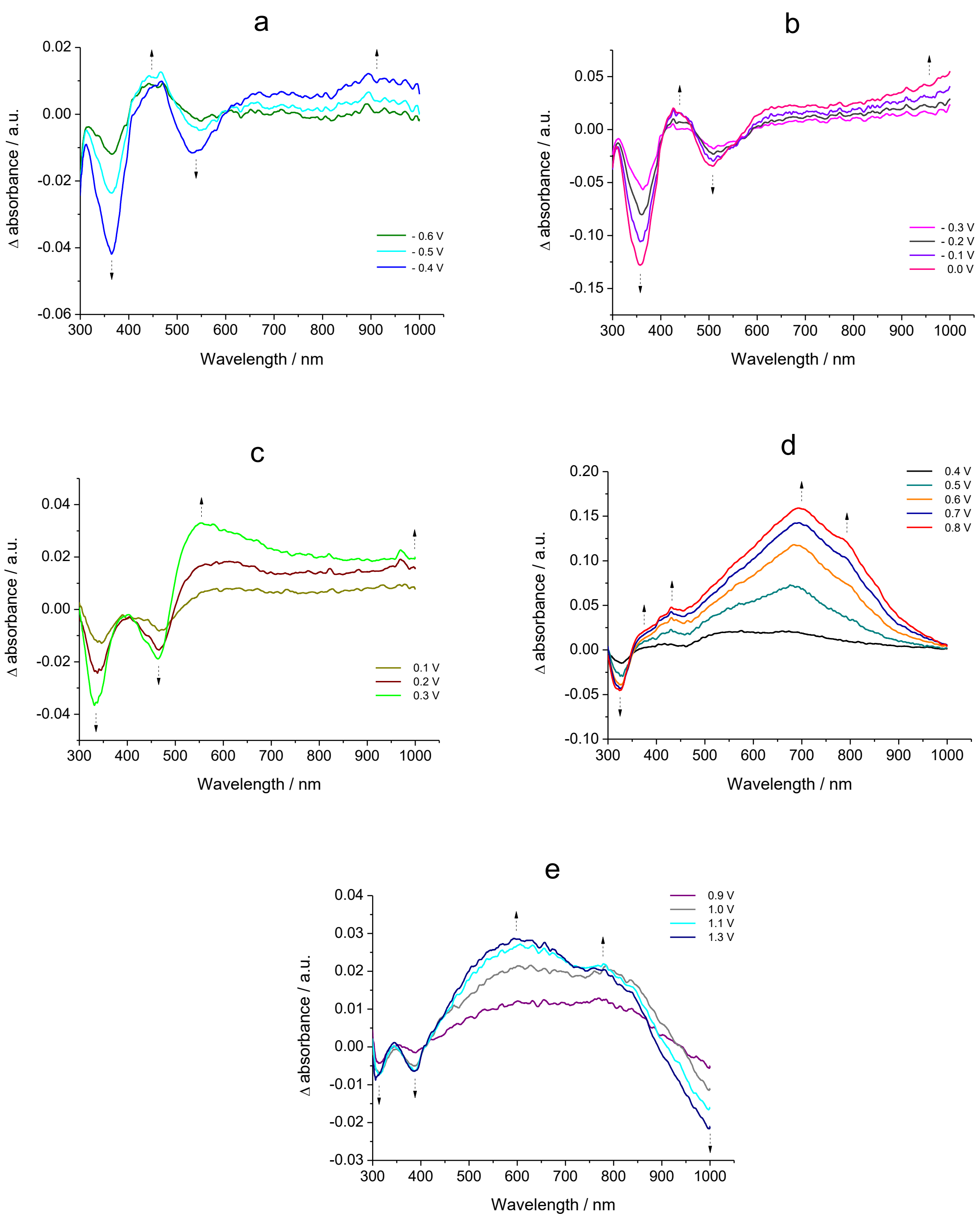

Figure S6. Differential UV-Vis spectra of a poly-[NiAmben]-modified ITO-coated glass electrode during redox switching in 0.05 M Et_4NBF_4/DCE: (**a**) spectra from −0.8 to −0.4 V, referenced to the spectrum of the polymer at −0.9 V; (**b**) spectra from −0.3 to 0.0 V, referenced to the spectrum of the polymer at −0.4 V; (**c**) spectra from 0.1 to 0.3 V, referenced to the spectrum of the polymer at 0.0 V; (**d**) spectra from 0.4 to 0.8 V, referenced to the spectrum of the polymer at 0.3 V; (**e**) spectra from 0.9 to 1.3 V, referenced to the spectrum of the polymer at 0.8 V.

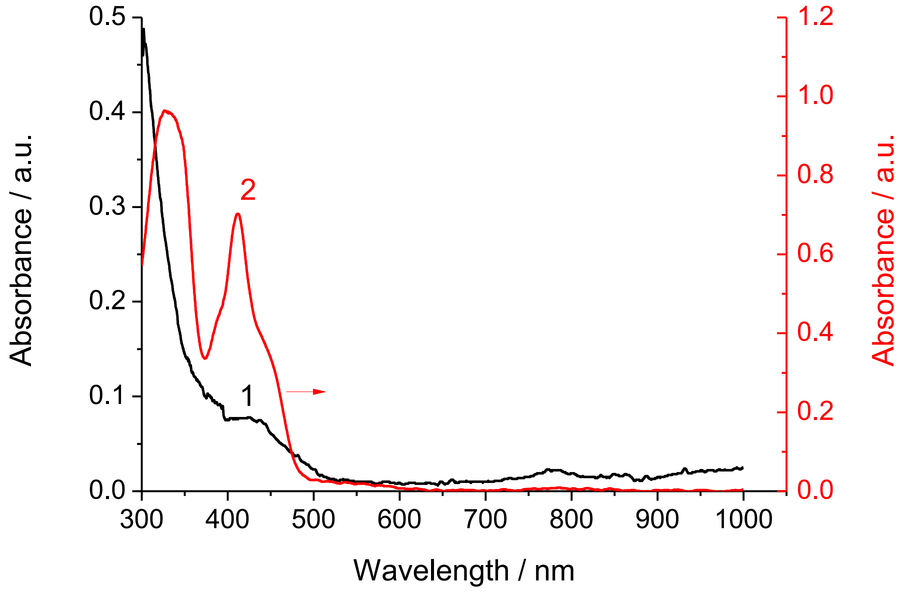

Figure S7. UV-Vis spectra of a poly-[NiSalen]-modified ITO-coated glass electrode at −0.3 V acquired in 0.05 M Et_4NBF_4/DCE (black curve 1) and the spectrum of [NiSalen] monomer (red curve 2).

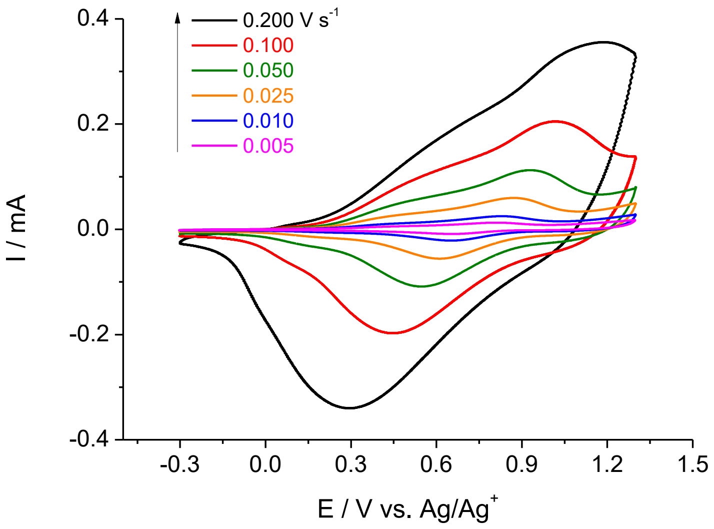

Figure S8. CV curves of a poly-[NiSalen]-modified Pt-coated quartz crystal electrode (1.37 cm^2) in 0.05 M Et_4NBF_4/DCE between −0.3 and 1.3 V at different scan rates (0.005–0.2 V s^{-1}).

Table S3. Coulombic efficiency of poly-[NiSalen] in the potential range between −0.3 and 1.3 V in 0.05 M Et_4NBF_4/DCE at different scan rates (calculated from the CV data shown in Figure S8).

Scan rate, v_s (V s^{-1})	Injected (oxidation) charge, Q_{ox} (mC)	Recovered (reduction) charge, Q_{red} (mC)	Coulombic efficiency, Q_{red} / Q_{ox}
0.005	2.09	0.91	0.44
0.01	1.86	1.10	0.59
0.025	1.66	1.21	0.73
0.05	1.55	1.29	0.83
0.1	1.46	1.29	0.88
0.2	1.32	1.23	0.93

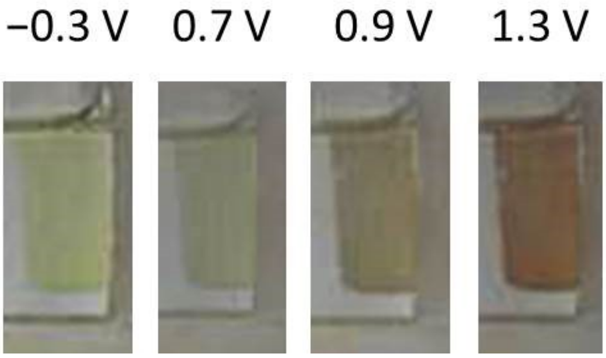

Figure S9. The color change of the poly-[NiSalen] film with varying potentials.
